# Effect of a Coordinated Community and Chronic Care Model Team Intervention vs Usual Care on Systolic Blood Pressure in Patients With Stroke or Transient Ischemic Attack: The SUCCEED Randomized Clinical Trial

**DOI:** 10.1001/jamanetworkopen.2020.36227

**Published:** 2021-02-15

**Authors:** Amytis Towfighi, Eric M. Cheng, Monica Ayala-Rivera, Frances Barry, Heather McCreath, David A. Ganz, Martin L. Lee, Nerses Sanossian, Bijal Mehta, Tara Dutta, Ali Razmara, Robert Bryg, Shlee S. Song, Phyllis Willis, Shinyi Wu, Magaly Ramirez, Adam Richards, Nicholas Jackson, Jeremy Wacksman, Brian Mittman, Jamie Tran, Renee R. Johnson, Chris Ediss, Theresa Sivers-Teixeira, Betty Shaby, Ana L. Montoya, Marilyn Corrales, Elizabeth Mojarro-Huang, Marissa Castro, Patricia Gomez, Cynthia Muñoz, Diamond Garcia, Lilian Moreno, Maura Fernandez, Enrique Lopez, Sarah Valdez, Hilary R. Haber, Valerie A. Hill, Neal M. Rao, Beatrice Martinez, Lillie Hudson, Natalie P. Valle, Barbara G. Vickrey

**Affiliations:** 1 University of Southern California, Los Angeles; 2 Los Angeles County Department of Health Services, Los Angeles, California; 3 Rancho Los Amigos National Rehabilitation Center, Downey, California; 4 LAC+USC Medical Center, Los Angeles, California; 5 University of California, Los Angeles; 6 Veterans Affairs Greater Los Angeles Healthcare System, Los Angeles, California; 7 Kaiser Permanente, Los Angeles, California; 8 Harbor-UCLA Medical Center, Torrance, California; 9 University of Maryland, Baltimore; 10 Kaiser Permanente, Irvine, California; 11 Olive View-UCLA Medical Center, Sylmar, California; 12 Cedars Sinai Medical Center, Los Angeles, California; 13 Watts Labor Community Action Committee, Los Angeles, California; 14 University of Washington School of Public Health, Seattle; 15 Community Partners International, San Francisco, California; 16 Dimagi, Cambridge, Massachusetts; 17 California State University, Los Angeles; 18 University of California, Riverside; 19 Brigham and Women’s Hospital, Boston, Massachusetts; 20 University of Cincinnati, Cincinnati, Ohio; 21 St Jude Medical Center, Fullerton, California; 22 Icahn School of Medicine at Mount Sinai, New York, New York

## Abstract

**Importance:**

Few stroke survivors meet recommended cardiovascular goals, particularly among racial/ethnic minority populations, such as Black or Hispanic individuals, or socioeconomically disadvantaged populations.

**Objective:**

To determine if a chronic care model–based, community health worker (CHW), advanced practice clinician (APC; including nurse practitioners or physician assistants), and physician team intervention improves risk factor control after stroke in a safety-net setting (ie, health care setting where all individuals receive care, regardless of health insurance status or ability to pay).

**Design, Setting, and Participants:**

This randomized clinical trial included participants recruited from 5 hospitals serving low-income populations in Los Angeles County, California, as part of the Secondary Stroke Prevention by Uniting Community and Chronic Care Model Teams Early to End Disparities (SUCCEED) clinical trial. Inclusion criteria were age 40 years or older; experience of ischemic or hemorrhagic stroke or transient ischemic attack (TIA) no more than 90 days prior; systolic blood pressure (BP) of 130 mm Hg or greater or 120 to 130 mm Hg with history of hypertension or using hypertensive medications; and English or Spanish language proficiency. The exclusion criterion was inability to consent. Among 887 individuals screened for eligibility, 542 individuals were eligible, and 487 individuals were enrolled and randomized, stratified by stroke type (ischemic or TIA vs hemorrhagic), language (English vs Spanish), and site to usual care vs intervention in a 1:1 fashion. The study was conducted from February 2014 to September 2018, and data were analyzed from October 2018 to November 2020.

**Interventions:**

Participants randomized to intervention were offered a multimodal coordinated care intervention, including hypothesized core components (ie, ≥3 APC clinic visits, ≥3 CHW home visits, and Chronic Disease Self-Management Program workshops), and additional telephone visits, protocol-driven risk factor management, culturally and linguistically tailored education materials, and self-management tools. Participants randomized to the control group received usual care, which varied by site but frequently included a free BP monitor, self-management tools, and linguistically tailored information materials.

**Main Outcomes and Measures:**

The primary outcome was change in systolic BP at 12 months. Secondary outcomes were non–high density lipoprotein cholesterol, hemoglobin A_1c_, and C-reactive protein (CRP) levels, body mass index, antithrombotic adherence, physical activity level, diet, and smoking status at 12 months. Potential mediators assessed included access to care, health and stroke literacy, self-efficacy, perceptions of care, and BP monitor use.

**Results:**

Among 487 participants included, the mean (SD) age was 57.1 (8.9) years; 317 (65.1%) were men, and 347 participants (71.3%) were Hispanic, 87 participants (18.3%) were Black, and 30 participants (6.3%) were Asian. A total of 246 participants were randomized to usual care, and 241 participants were randomized to the intervention. Mean (SD) systolic BP improved from 143 (17) mm Hg at baseline to 133 (20) mm Hg at 12 months in the intervention group and from 146 (19) mm Hg at baseline to 137 (22) mm Hg at 12 months in the usual care group, with no significant differences in the change between groups. Compared with the control group, participants in the intervention group had greater improvements in self-reported salt intake (difference, 15.4 [95% CI, 4.4 to 26.0]; *P* = .004) and serum CRP level (difference in log CRP, −0.4 [95% CI, −0.7 to −0.1] mg/dL; *P* = .003); there were no differences in other secondary outcomes. Although 216 participants (89.6%) in the intervention group received some of the 3 core components, only 35 participants (14.5%) received the intended full dose.

**Conclusions and Relevance:**

This randomized clinical trial of a complex multilevel, multimodal intervention did not find vascular risk factor improvements beyond that of usual care; however, further studies may consider testing the SUCCEED intervention with modifications to enhance implementation and participant engagement.

**Trial Registration:**

ClinicalTrials.gov Identifier: NCT01763203

## Key Points

**Question:**

Is a team-based community health worker and advanced practice clinician (including nurse practitioners or physician assistants) intervention emphasizing evidence-based care, self-management, lifestyle change, and medication adherence superior to usual care for controlling blood pressure after stroke in safety-net settings?

**Findings:**

In this randomized clinical trial that included 487 adults with recent stroke or transient ischemic attack, there was no difference between usual care and the multifaceted team-based intervention in blood pressure control at 12 months.

**Meaning:**

These findings suggest that additional research is needed to determine the optimal care model for controlling risk factors after stroke in safety-net settings.

## Teaser

This randomized clinical trial examines the effect of a multifaceted team intervention vs usual care on systolic blood pressure (SBP) among patients with stroke or transient ischemic attack (TIA) in safety-net settings.

## Introduction

Approximately 1 in 4 strokes in the United States are recurrent.^1^ Combining dietary changes, physical activity, and targeted medications can reduce the cumulative risk for recurrent vascular events after stroke by 80%.^2^ Nevertheless, risk factor control among survivors of stroke remains poor, with only a small portion of individuals reaching recommended targets.^3,4,5,6^ Independent factors associated with poor risk factor control include minority race/ethnicity (eg, Black or Hispanic), poverty, and lower education level.^4,5,6^

The chronic care model (CCM), which incorporates self-management support, delivery system redesign, clinical information systems with decision support for applying evidence-based care guidelines, health care system leadership engagement, and community resources, has been effective in improving outcomes while reducing costs for chronic conditions.^7,8,9,10^ A randomized clinical trial of a CCM-based intervention by Cheng et al^11^ did not show a benefit beyond usual care in blood pressure (BP) reduction in a predominantly Hispanic population treated for stroke in a safety-net setting (ie, health care setting where all individuals receive care, regardless of health insurance status or ability to pay). The intervention, delivered solely within the health care system by advanced practice clinicians (APCs; including nurse practitioners or physician assistants), did not address home and community barriers to lifestyle change or transportation barriers to accessing care.

We hypothesized that community health workers (CHWs) could more effectively address lifestyle factors, health literacy, medication adherence, and obstacles to behavior change. Therefore, we developed a multilevel, multicomponent, complex CCM-based team (including an APC, a CHW, and a physician) intervention for improving poststroke risk factor control.^12^ The intervention redesigned care at the health care system level with evidence-based care protocols with real-time electronic decision support and enhanced care coordination and at the patient level by targeting health and stroke literacy, medication adherence, self-management skills, and lifestyle (eFigure in Supplement 1). We tested the efficacy of the Secondary Stroke Prevention by Uniting Community and Chronic Care Model Teams Early to End Disparities (SUCCEED) intervention in improving risk factor control after stroke at 12 months in a safety-net setting.

## Methods

Institutional review board approvals were obtained at University of California, Los Angeles and at each of the 5 sites (or through reliance agreements with UCLA). All participants provided written informed consent. This study is reported following the Consolidated Standards of Reporting Trials (CONSORT) reporting guideline. The Trial Protocol is presented in Supplement 2.

### Setting and Participants

**Figure 1.**
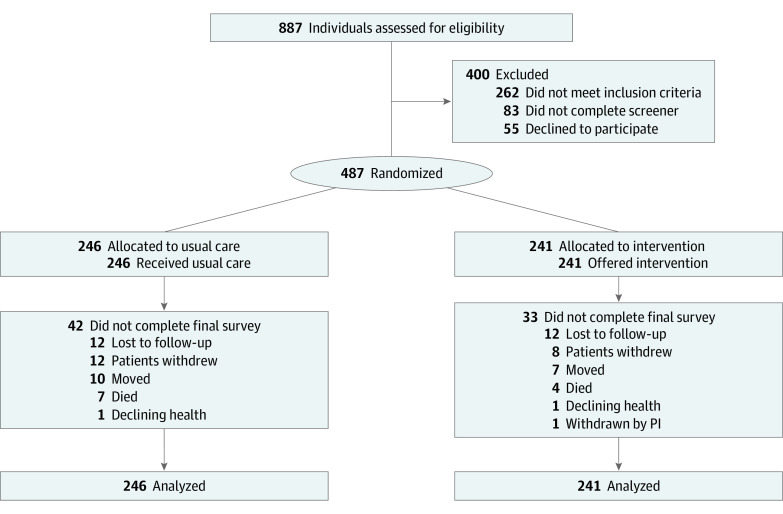
Consort Flow Diagram of the SUCCEED Trial PI indicates principal investigator; SUCCEED, Secondary Stroke Prevention by Uniting Community and Chronic Care Model Teams Early to End Disparities.

Study details have been previously published.^12^ The intervention was framed by the Health Belief Model.^13^ Briefly, of 887 screened participants, 542 were eligible, and 487 were enrolled from February 2014 through August 2017 from 4 Los Angeles County Department of Health Services public safety-net health care system hospitals (ie, Rancho Los Amigos National Rehabilitation Center, Harbor-UCLA, LAC+USC Medical Center, and Olive View Medical Center) and 1 additional hospital (ie, Cedars Sinai) serving low-income zip codes (Figure 1). Inclusion criteria were age 40 years or older; experience of transient ischemic attack (TIA), ischemic stroke, or intracerebral hemorrhage within the last 90 days; and elevated systolic BP. Elevated systolic BP was defined as greater than 120 mm Hg, consistent with existing guidelines.^14^ During the course of the trial, evidence of a potential J-shaped curve emerged among individuals who survived stroke, with a suggestion of higher risk for cardiovascular events with strict BP control.^15,16,17,18^ Therefore, 16 months after initiating the trial (after enrolling 154 participants), the systolic BP goal at 12 months was increased from less than 120 mm Hg to less than 130 mm Hg. Correspondingly, inclusion criteria were revised to systolic BP 130 mm Hg or greater or 120 to 130 mm Hg in individuals with a history of hypertension or using antihypertensive medications prior to the stroke or TIA. Participants were excluded if they were unable to communicate understanding the study during the informed consent process or lacked English or Spanish proficiency.

### Study Procedures

After baseline data collection, eligible participants were randomized 1:1 to control vs intervention, stratified by stroke type (TIA or ischemic vs hemorrhagic), language (English vs Spanish), and study site. Randomization schedules were developed using computer-assisted stratified randomization of a block size of 4 and programmed into the Research Electronic Data Capture (Vanderbilt University) database.^12^ Randomization schedules were only accessible to the project manager (M.A.-R.), principal investigators (A.T. and B.G.V.), and data manager (F.B.). Research assistants (C.M., D.G., L.M., M.F., E.L., and S.V.) obtaining outcome assessments were blinded to the randomization arm throughout follow-up and did not interact with care teams.^12^

### Usual Care and Intervention

Participants randomized to the control group received usual care, which varied by study site (eTable 1 in Supplement 1). Most study participants (325 participants [66.7%]) were enrolled at a site that provided free BP monitors, self-management tools (BP and glucose logs), and linguistically tailored educational materials as usual care.

In addition to usual care, individuals randomized to the intervention arm were offered at least 3 APC clinic visits; at least 3 CHW home visits; telephone visits; protocol-driven risk factor management; electronic decision support for clinicians; coordination of care; BP monitors (HEM-711 DLX; Omron Healthcare); culturally and linguistically tailored educational materials; vascular risk factor goal tools; and Chronic Disease Self-Management Program (CDSMP)^19^ workshops.^12^ Intervention participants received the BP monitors and educational materials immediately after randomization. A community advisory board offered input throughout intervention development and implementation. All care management tasks and protocols were programmed into a mobile- and web-based care management application (CommCare; Dimagi)^20^ that offered immediate access to patient-specific information, patient goals, real-time decision support, protocols, assessment tools, tasks, and educational materials.^12^ The CDSMP workshops were offered in English and Spanish by 2 CHWs at health care system and community locations convenient to participants (CHWs included A.L.M., M. Corrales, E.M.-H., M. Castro, and P.G). Each participant received a companion book in English^47^ or Spanish,^48^ and a relaxation CD in their preferred language. Care teams were encouraged to tailor the intervention to participants’ needs and assist participants in goal-setting. Participants could opt out of any aspect of the intervention.

The APCs (J.T., R.R.J., C.E., T.S.-T., B.S.) prescribed and titrated medications, emphasized medication adherence, and reinforced self-management skills. The CHWs assessed and addressed medication adherence, provided stroke education and self-management training, served as a liaison with the health care system, and offered resources or referrals to address social determinants of health.

The intervention was delivered in participants’ preferred language (English or Spanish). All CHWs were bilingual in English and Spanish. If APCs did not speak Spanish, clinic visits included an interpreter or CHW. All APCs and CHWs received extensive training.^12^

Process evaluations were conducted to evaluate implementation barriers. Care teams met weekly to review participants’ care plans. Additionally, study leaders (A.T., M.A.-R., and B.G.V.) met with care teams weekly to address intervention fidelity and address barriers.

### Outcome Measures

Outcomes were assessed in person at baseline, 3 months, and 12 months and via telephone at 8 months.^12^ The primary outcome was change in mean systolic BP at 12 months. Secondary outcomes included non–high density lipoprotein (HDL) cholesterol level, hemoglobin A_1c_ (HbA_1c_) level, C-reactive protein (CRP) level, body mass index, waist circumference, and self-reported physical activity,^21^ fruit and vegetable intake,^22^ soda consumption,^22^ salt intake,^23^ smoking status,^22^ and antithrombotic medication use (for participants with ischemic stroke and TIA).

Potential mediators included health literacy,^24^ stroke worry,^25^ perceived risk of stroke,^25^ stroke literacy,^26,27^ global medication adherence,^28^ individual medication adherence,^29^ BP monitor use, self-efficacy,^30^ intention to quit smoking, access to care,^31^ patient perceptions of care (assessed using the Patient Assessment of Chronic Illness Care^33^ and Consumer Assessment of Healthcare Providers and Systems^32^), depression (assessed using the Patient Health Questionnaire 9^34^), health care utilization,^35^ social support,^36^ and health-related quality of life (assessed using Short Form 6 Dimension^37^). Potential mediators for the intervention included number of clinic visits, number of home visits, and participation in CDSMP workshops.

Potential moderators included sex, self-reported race and ethnicity, country of birth (classified as US vs non-US), preferred language (English vs Spanish), education, employment prior to stroke, insurance, marital status, living situation, study site, type of cerebrovascular event (classified as ischemic stroke or TIA vs intracerebral hemorrhage), stroke severity (assessed using the National Institutes of Health Stroke Scale), functional status (assessed using the modified Rankin Scale score), number of comorbidities, primary care clinician, chaos,^38^ competing needs,^31^ and acculturation.^39^ Chaos was measured using a brief measure of global life chaos in adults that includes questions regarding the extent to which one’s life is organized, predictable, and stable.^38^

### Analytical Sample Size, Statistical Power, and Enrollment Sample Size

Sample size calculation and power analyses were based on systolic BP as the primary outcome. A small effect size for systolic BP (0.25 in SD units or 5.06 mm Hg) can be considered clinically meaningful in the presence of potential moderators and mediators. Power analyses were conducted for comparison of systolic BP between intervention and control arms. Using intraclass correlation of the 5 sites at 0.0085 level, SD of 20 mm Hg, 3 repeated measurements, type I error of 0.05, type II error of 0.2 (equivalent to power of 80%), 2.4 mean data points for each participant (corresponding to 30% attrition), and an autocorrelation of 0.2, the effective minimum sample size for the entire study was 261 (after adjusting for clustering effect of the 5 recruitment sites).

### Statistical Analysis

Data analysis was performed from October 2018 to November 2020. Comparisons of baseline characteristics between usual care and intervention arms and between participants who completed the 12-month survey vs those who did not were conducted using the 2-sample *t* test, χ^2^ test, Fisher exact test, or Wilcoxon rank-sum test, depending on the nature of the data.

Outcomes were compared using baseline and 12-month time points and using repeated-measures mixed-effects models that included baseline, 3-month, 8-month (only for knowledge of stroke factors and medication adherence), and 12-month data. All analyses used an intent-to-treat approach. Stratification variables (ie, site, language, stroke type) were included in repeated measures models as covariates. The key analysis was interaction of time and study arm. The bootstrap method was used to calculate 95% CIs and 2-sided *P* values for some variables. A 5% level of significance was used throughout.

Four sensitivity analyses were performed for the repeated measures models (eAppendix in Supplement 1); 2 of these sensitivity models included attrition weights determined by a logistic model with survey language, age, and marital status as predictor variables. In addition, an analysis of primary and secondary outcomes with an indicator variable for the ordinal categories of implementation (as a covariate) by dose was conducted. Intervention participation was classified into 5 categories a priori: (1) 3 or more clinic visits, 3 or more home visits, and 4 or more CDSMP classes; (2) 3 or more clinic visits, 3 or more home visits, and fewer than 4 CDSMP classes; (3) 2 or more clinic visits and 2 or more home visits; (4) fewer than 2 clinic visits and fewer than 2 home visits; and (5) no intervention.

For participants randomized to the intervention, a multivariable analysis evaluated the independent association of each core component (clinic visit, home visit, and CDSMP) on 12-month change in BP. Covariates included site, stroke type (ischemic stroke or TIA vs ICH) and language. Core components were dichotomized: 3 or more vs fewer than 3 clinic visits; 3 or more vs fewer than 3 home visits, and 4 or more vs fewer than 4 CDSMP classes.

Among participants with ischemic stroke, the relative risk reduction (RRR) of recurrent stroke achieved in each study arm and modified Global Outcome Score (representing the proportion of potentially preventable stroke risk reduction achieved with the level of care provided at the end of the trial, given the level of care received at the beginning of the trial) were calculated (eAppendix in Supplement 1).^40^

## Results

Of 487 enrolled participants, the mean (SD) age was 57.1 (8.9) years; 317 participants (65.1%) were men, and 347 participants (71.3%) were Hispanic, 87 (18.3%) were Black, and 30 (6.3%) were of Asian descent (Table 1). Most participants (383 participants [78.6%]) had ischemic stroke. Although 304 participants (62.4%) had mild strokes (ie, National Institutes of Health Stroke Scale score ≤5), 310 participants (63.7%) had at least moderate disability (ie, modified Rankin Scale score ≥3). Only 135 participants (27.7%) were born in the United States; 298 of 482 participants (61.8%) had not graduated high school (Table 1). A total of 308 participants (69.1%) had government insurance (Medicaid or Medicare), and 93 participants (20.9%) were uninsured. Participants had moderate levels of life chaos, and up to one-quarter reported competing subsistence needs (eTable 2 in Supplement 1).

**Table 1.** Sociodemographic and Clinical Patient Characteristics

Variable	Participants, No. (%)		
	Total (n = 487)	Usual care (n = 246)	Intervention (n = 241)
Site			
Rancho Los Amigos National Rehabilitation Center	325 (66.7)	162 (65.9)	163 (67.6)
Harbor-UCLA Medical Center	74 (15.2)	37 (15.0)	37 (15.4)
LAC+USC Medical Center	67 (13.8)	34 (13.8)	33 (13.7)
Olive View-UCLA Medical Center	6 (1.2)	4 (1.6)	2 (0.8)
Cedars Sinai Medical Center	15 (3.1)	9 (3.7)	6 (2.5)
Language			
English	207 (42.5)	106 (43.1)	101 (41.9)
Spanish	280 (57.5)	140 (56.9)	140 (58.1)
Index event			
Ischemic stroke	383 (78.6)	193 (78.5)	187 (77.6)
Intracerebral hemorrhage	78 (16.0)	38 (15.4)	43 (17.8)
TIA	26 (5.3)	15 (6.1)	11 (4.6)
NIHSS			
Mild (≤5)	304 (62.4)	159 (64.6)	145 (60.2)
Moderate (6-14)	172 (35.3)	81 (32.9)	91 (37.8)
Severe (15-24)	11 (2.3)	6 (2.4)	5 (2.1)
Very severe (>24)	0	0	0
Modified Rankin Scale			
No disability (0)	30 (6.2)	18 (7.3)	12 (5.0)
Not significant (1)	66 (13.6)	33 (13.4)	33 (13.7)
Slight (2)	81 (16.6)	49 (19.9)	32 (13.3)
Moderate (3)	72 (14.8)	31 (12.6)	41 (17.0)
Moderate/severe (4)	137 (28.1)	64 (26.0)	73 (30.3)
Severe (5)	101 (20.7)	51 (20.7)	50 (20.7)
Age, mean (SD), y	57.1 (8.9)	57 (8.7)	57.2 (9.0)
Sex			
Women	170 (34.9)	92 (37.4)	78 (32.4)
Men	317 (65.1)	154 (62.6)	163 (67.6)
Race			
White	335 (70.4)	167 (69.9)	168 (70.9)
Black	87 (18.3)	42 (17.6)	45 (19.0)
Asian	30 (6.3)	15 (6.3)	15 (6.3)
≥1 Race	10 (2.1)	7 (2.9)	3 (1.3)
Native American or Alaskan Native	9 (1.9)	4 (1.7)	5 (2.1)
Native Hawaiian or other Pacific Islander	5 (1.1)	4 (1.7)	1 (0.4)
Hispanic ethnicity	347 (71.3)	177 (72.0)	170 (70.5)
Born outside the United States	352 (72.4)	176 (71.8)	176 (73.0)
Educated in the United States	3 (3.6)	3 (1.7)	0
Time in United States, mean (SD), y	27.9 (12.15)	27.3 (11.6)	28.4 (12.7)
Living with ≥1 other adult	430 (88.7)	219 (89.4)	211 (87.9)
Education			
Some college	147 (30.5)	73 (29.9)	74 (31.1)
High school graduate or GED	37 (7.7)	22 (9.6)	15 (6.6)
Some high school but did not graduate	115 (23.9)	56 (24.3)	59 (26.1)
≤Eighth Grade	183 (38.0)	93 (38.1)	90 (37.8)
Working for pay prior to stroke	266 (55.1)	141 (58.0)	125 (52.1)
Married or in domestic partnership	219 (45.1)	114 (46.5)	105 (43.6)
Insurance			
Government	308 (69.1)	157 (70.4)	151 (67.7)
Private insurance (with or without government)	45 (10.1)	21 (9.4)	24 (10.8)
None	93 (20.9)	45 (20.2)	48 (21.5)
Has primary care clinician	231 (47.7)	120 (49.4)	111 (46.1)
Living situation			
Own home	338 (71.9)	167 (71.4)	171 (72.5)
Shelter or street	6 (1.3)	3 (1.3)	3 (1.3)
Nursing, group home, or board and care	2 (0.4)	2 (0.9)	0
With relative	93 (19.8)	46 (19.7)	47 (19.9)
Room in house or hotel	31 (6.6)	16 (6.8)	15 (6.4)

Abbreviations: GED, general educational development; NIHSS, National Institutes of Health Stroke Scale; TIA, transient ischemic attack.

Intervention and usual care groups were not significantly different with respect to baseline sociodemographic characteristics (Table 1); however, the intervention arm had a lower percentage of participants watching or reducing their salt intake and higher log CRP at baseline (eTable 3 in Supplement 1).

A total of 412 participants (84.6%) completed the 12-month visit. At follow-up, there were no differences in the mean systolic BP improvement nor in the proportion achieving systolic BP control across arms, though systolic BP improved within each arm (Table 2). From baseline to 12 months, mean (SD) systolic BP improved from 143 (17) mm Hg to 133 (20) mm Hg among participants in the intervention group and from 146 (19) mm Hg to 137 (22) mm Hg among participants in the usual care group (between-group difference, −3.3 [95% CI, −14.9 to 8.8] mm Hg; *P* = .57) (Table 2). Controlled systolic BP (ie, <130 mm Hg) increased from 62 participants (25.7%) at baseline to 92 participants (44.7%) at 12 months in the intervention group and 53 participants (21.5%) to 89 participants (43.6%) in the usual care group).

**Table 2.** Primary and Secondary Outcomes

Outcome	Usual care			Intervention			Differences in changes from baseline between usual care and intervention (95% CI)	*P* value
	Patients, No.	Mean (SD)	Change from baseline (95% CI)	Patients, No.	Mean (SD)	Change from baseline (95% CI)		
**Primary**								
SBP ≤130 mm Hg, No. (%)								
Baseline	246	53 (21.5)	22.1 (13.9 to 30.3)^a^	241	62 (25.7)	18.9 (10.7 to 27.1)^a^	−3.3 (−14.9 to 8.8)	.57
12 mo	204	89 (43.6)		206	92 (44.7)			
SBP, mm Hg								
Baseline	246	145.7 (18.6)	−8.1 (−11.4 to −4.8)^a^	241	143.2 (17.1)	−9.8 (−13.1 to −6.5)^a^	−1.7 (−6.4 to 2.9)	.46
12 mo	204	136.9 (21.9)		206	133.1 (20.5)			
**Secondary**								
Non-HDL, mg/dL								
Baseline	201	95.7 (40.4)	6.1 (−2.3 to 14.5)	191	94.1 (42.3)	2.8 (−5.2 to 10.8)	−3.3 (−14.9 to 8.3)	.57
12 mo	197	104.1 (49.2)		202	98.7 (47.8)			
HbA_1c_, %								
Baseline	160	6.8 (1.4)	−0.2 (−0.4 to 0.1)	161	6.8 (1.6)	−0.3 (−0.6 to −0.1)^a^	−0.2 (−0.5 to 0.2)	.36
12 mo	144	6.6 (1.3)		161	6.5 (1.2)			
Log CRP, mg/dL								
Baseline	198	−1.7 (−1.2)	−0.4 (−0.6 to −0.2)^a^	202	−1.3 (−1.2)	−0.8 (−1.1 to −0.6)^a^	−0.4 (−0.7 to −0.1)	.003
12 mo	180	−2.1 (−1.2)		192	−2.2 (−1.2)			
BMI								
Baseline	173	29.1 (5.1)	0.4 (−0.1 to 0.8)	163	29.2 (5.7)	0.6 (0.1 to 1.1)^a^	0.2 (−0.4 to 0.9)	.53
12 mo	182	29.2 (5.01)		186	29.5 (5.9)			
Physical activity, IPAQ, MET min/wk, median (IQR)								
Baseline	244	540 (0 to 2040)	20 (−240 to 360)	239	600 (40 to 2400)	40 (−240 to 240)	20 (−400 to 300)	.93
12 mo	203	560 (0 to 1680)		205	640 (160 to 1680)			
≥5 daily servings of fruit/vegetables, No. (%)								
Baseline	244	30 (12.3)	1.7 (−4.2 to 7.6)	239	20 (8.4)	2.3 (−3.2 to 7.8)	0.7 (−7.7 to 8.7)	.84
12 mo	200	28 (14.0)		206	22 (10.7)			
0 daily servings of soda, No. (%)								
Baseline	245	69 (28.2)	22.6 (15.0 to 30.2)^a^	240	69 (28.8)	23.2 (15.4 to 31.0)^a^	0.7 (−10.6 to 12.1)	.86
12 mo	201	102 (50.7)		206	107 (51.9)			
Reducing or monitoring salt intake, No. (%)								
Baseline	245	134 (54.7)	28.5 (20.8 to 36.2)^a^	240	104 (43.3)	43.7 (36.7 to 50.7)^a^	15.4 (4.4 to 26.0)	.004
12 mo	202	168 (83.2)		208	181 (87.0)			
Not smoking, No. (%)								
Baseline	243	192 (79.0)	10.2 (5.8 to 14.5)	240	183 (76.3)	11.7 (6.8 to 16.5)	1.6 (−4.8 to 8)	.66
12 mo	203	181 (89.2)		207	182 (87.9)			
Using antithrombotic medication, No. (%)^b^								
Baseline	206	205 (99.5)	−7.8 (−11.8 to −3.8)	199	197 (99.0)	−8.4 (−12.7 to −4.0)	−0.6 (−6.7 to 5.3)	.80
12 mo	169	155 (91.7)		171	155 (90.6)			

Abbreviations: BMI, body mass index; CRP, C-reactive protein; HbA_1c_, hemoglobin A_1c_; HDL, high density lipoprotein cholesterol; IPAQ, International Physical Activity Questionnaire; SBP, systolic blood pressure.

SI conversion factors: To convert CRP to milligrams per liter, multiply by 10; cholesterol to millimoles per liter, multiply by 0.029; hemoglobin to proportion of total hemoglobin, multiply by 0.01.

^a^ 95% confidence interval does not contain zero.

^b^ Among patients with transient ischemic attack or ischemic stroke.

At 12 months, the intervention participants had a larger improvement in self-reported reduction of salt intake than usual care (between-group difference in the proportion who reported they had reduced their salt intake, 15.4 [95% CI, 4.4 to 26.0]; *P* = .004) (Table 2). Intervention and usual care groups did not differ on changes in the other secondary outcomes. Mean CRP decreased in both groups, but there was a larger decrease in the intervention group (control: change in log CRP, −0.4 [95% CI, −0.6 to −0.2] mg/dL; intervention: change, −0.8 [95% CI, −1.1 to −0.6] mg/dL; between group difference, −0.4 [95% CI, −0.7 to −0.1] mg/dL; *P* = .003) (to convert to milligrams per liter, multiply by 10). A total of 314 participants (64.5%) had missing waist circumference at baseline because they could not stand for measurements (eTable 3 in Supplement 1); the proportions missing were so large that the results were not considered valid. The 4 repeated measures sensitivity analyses did not differ from the main analysis for any of the primary or secondary outcome measures.

Regarding factors associated with mediating outcomes, compared with usual care, at 12 months, participants in the intervention group had a greater increase in BP monitor use, number of appointments with APCs, and statin use (for participants with ischemic stroke or TIA) (Table 3). There were no differences between groups over time for other factors associated with mediation.

**Table 3.** Potential Factors Associated With Mediation of Outcomes

Outcome	Usual Care			Intervention			Differences in changes from baseline between usual care and intervention (95% CI)	*P* value
	Patients, No.	Mean (SD)	Change from baseline	Patients, No.	Mean (SD)	Change from baseline		
Health literacy								
Confidence filling out medical forms on own^a^								
Baseline	242	4 (1.3)	−1 (−1.3 to −0.8)^b^	240	3.8 (1.5)	−0.9 (−1.2 to −0.7)^b^	0.1 (−0.2 to 0.5)	.44
12 mo	201	2.9 (1.6)		208	2.8 (1.5)			
Frequency of problems learning about medical condition owing to difficulty understanding written information^c^								
Baseline	237	3.8 (1.4)	−0.3 (−0.5 to −0.1)^b^	235	3.6 (1.5)	−0.2 (−0.4 to 0.1)	0.1 (−0.2 to 0.5)	.41
12 mo	203	3.5 (1.4)		208	3.4 (1.4)			
Frequency of having someone help read clinic or hospital materials^c^								
Baseline	242	3.8 (1.5)	−1.1 (−1.4 to −0.8)^b^	236	3.7 (1.6)	−1.2 (−1.5 to −0.9)^b^	−0.1 (−0.5 to 0.3)	.50
12 mo	203	2.8 (1.6)		208	2.5 (1.5)			
Frequency of having problems understanding what was told about medical condition^c^								
Baseline	239	4 (1.4)	−0.3 (−0.6 to −0.1)^b^	236	4 (1.4)	−0.3 (−0.6 to 0)	0 (−0.3 to 0.4)	.87
12 mo	203	3.7 (1.4)		207	3.6 (1.3)			
Worried about having another stroke, No. (%)								
Baseline	243	66 (27.2)	17.7 (9.9 to 25.4)^b^	240	68 (28.3)	19.0 (10.7 to 27.3)^b^	1.4 (−10.0 to 12.9)	.82
12 mo	203	91 (44.8)		207	98 (47.3)			
Believe they have a higher likelihood of having another stroke, No. (%)								
Baseline	243	140 (57.6)	−19.1 (−10.3 to −27.9)^b^	240	126 (53.2)	−14.9 (−6.5 to −23.3)^b^	−4.1 (−16.5 to 8.5)	.50
12 mo	203	77 (38.5)		207	77 (38.3)			
Stroke literacy								
Knows ≥1 stroke risk factors, No. (%)								
Baseline	246	208 (84.6)	2.7 (−3.2 to 8.6)	241	196 (81.3)	1.4 (−4.5 to 7.2)	−1.5 (−10.3 to 7.1)	.62
12 mo	204	178 (87.3)		208	172 (82.7)			
Knows ≥3 stroke risk factors, No. (%)								
Baseline	246	66 (26.8)	−2.8 (−10.4 to 4.7)	241	55 (22.8)	3.1 (−4.2 to 10.4)	6.1 (−5.0,16.6)	.26
12 mo	204	49 (24.0)		208	54 (26.0)			
Names BP as a stroke risk factor, No. (%)								
Baseline	246	101 (41.1)	−3.8 (−11.6 to 4.0)	241	91 (37.8)	5.0 (−3.3 to 13.4)	9.0 (−3.0 to 20.5)	.11
12 mo	204	76 (37.3)		208	89 (42.8)			
Medication adherence								
Adheres to medications^d^								
Baseline	152	3.7 (1.5)	1.0 (0.7 to 1.3)^b^	155	3.5 (1.58)	1.1 (0.8 to 1.4)^b^	0.1 (−0.3 to 0.5)	.68
12 mo	191	4.6 (0.9)		195	4.6 (0.96)			
Prescribed and filled prescription for antihypertensive, No. (%)								
Baseline	244	219 (89.8)	0.4 (−4.0 to 4.9)	238	204 (85.7)	5.2 (−0.3 to 10.6)	4.6 (−2.2 to 11.4)	.21
12 mo	204	184 (90.2)		208	189 (90.9)			
Prescribed and filled prescription for statin medication, No. (%)^e^								
Baseline	207	196 (94.7)	−13.4 (−19.3 to −7.5)	196	179 (91.3)	−4.9 (−11.2 to 1.5)	8.5 (0.4 to 16.9)	.04
12 mo	171	139 (81.3)		170	147 (86.5)			
Prescribed and filled prescription for antidepressant medication, No. (%)								
Baseline	244	38 (15.6)	−2.3 (−7.5 to 2.9)	237	46 (19.4)	−4.5 (−10.3 to 1.2)	−2.1 (−9.9 to 5.7)	.56
12 mo	204	27 (13.2)		208	31 (14.9)			
BP monitor use								
Use a BP monitor at home, No. (%)								
Baseline	246	62 (25.2)	45.2 (37.4 to 53.1)^b^	240	63 (26.3)	63.2 (56.2 to 70.1)^b^	17.7 (7.0 to 28.1)	<.001
12 mo	203	143 (70.4)		208	186 (89.4)			
Use a BP monitor ≥1 time/d, No. (%)								
Baseline	246	20 (8.1)	30.8 (23.3 to 38.2)^b^	239	22 (9.2)	53.3 (45.7 to 60.9)^b^	22.1 (11.8 to 32.2)	<.001
12 mo	203	79 (38.9)		208	130 (62.5)			
Self-efficacy^f^								
Baseline	245	30.9 (7.9)	0.5 (−0.8 to 1.8)	239	31.8 (6.9)	0.2 (−1.0 to 1.3)	−0.3 (−2.0 to 1.4)	.72
12 mo	201	31.6 (8.1)		207	31.3 (7.6)			
Intend to quit smoking in next month or already quit smoking, No. (%)								
Baseline	51	30 (58.8)	−2.4 (−18.1 to 13.3)	57	32 (56.1)	−7.7 (−9.4 to 24.8)	8.1 (−11.7 to 28.9)	.43
12 mo	39	22 (56.4)		47	30 (63.8)			
Access to care								
If I need hospital care, I can get admitted without any trouble^g^								
Baseline	246	1.6 (1.1)	−0.2 (−0.4 to 0)	239	1.6 (1.1)	−0.2 (−0.4 to 0)	0 (−0.2 to 0.3)	.91
12 mo	202	1.4 (0.9)		208	1.4 (0.9)			
It is hard for me to get medical care in an emergency^h^								
Baseline	246	3.7 (1.6)	0.3 (0.1 to 0.6)^b^	240	3.6 (1.7)	0.5 (0.2 to 0.8)^b^	0.2 (−0.2 to 0.5)	.42
12 mo	202	4 (1.5)		206	4 (1.6)			
Sometimes I go without the medical care needed because it is too expensive^h^								
Baseline	245	3 (1.7)	1.2 (0.9 to 1.4)^b^	238	2.9 (1.8)	1.1 (0.8 to 1.4)^b^	−0.1 (−0.5 to 0.3)	.73
12 mo	201	4.2 (1.4)		206	4 (1.6)			
I have easy access to the medical specialists I need^g^								
Baseline	243	2.3 (1.5)	−0.4 (−0.7 to −0.2)^b^	236	2.3 (1.5)	−0.5 (−0.8 to −0.3)^b^	−0.1 (−0.4 to 0.3)	.67
12 mo	201	1.9 (1.3)		205	1.7 (1.3)			
Places where I can get medical care are very conveniently located^g^								
Baseline	245	1.9 (1.3)	−0.3 (−0.5 to −0.1)^b^	239	1.8 (1.3)	0.1 (−0.1 to 0.4)	0.4 (0.1 to 0.8)	.01
12 mo	202	1.6 (1.2)		206	1.9 (1.4)			
I am able to get medical care whenever I need it^g^								
Baseline	245	1.8 (1.3)	−0.4 (−0.6 to −0.2)^b^	239	1.8 (1.3)	−0.4 (−0.6 to −0.2)	0 (−0.3 to 0.3)	.95
12 mo	202	1.3 (0.9)		207	1.4 (1)			
Perceptions of care (PACIC)				96				
PACIC overall score^a^								
Baseline	96	2.5 (1.0)	0.3 (0.1 to 0.5)^b^	96	2.6 (1.1)	0.5 (0.3 to 0.7)^b^	0.2 (−0.1 to 0.5)	.25
12 mo	175	2.8 (1.1)		186	3.2 (1.1)			
Patient activation^a^								
Baseline	104	2.5 (1.2)	0.5 (0.2 to 0.8)^b^	101	2.7 (1.4)	0.5 (0.2 to 0.8)^b^	0 (−0.5 to 0.4)	.97
12 mo	183	3 (1.4)		189	3.2 (1.4)			
Decision support^a^								
Baseline	101	3.1 (1.2)	0.4 (0.1 to 0.7)^b^	101	3.2 (1.3)	0.6 (0.3 to 0.9)^b^	0.1 (−0.3 to 0.5)	.47
12 mo	184	3.5 (1.3)		191	3.8 (1.2)			
Goal setting^a^								
Baseline	101	2.6 (1.2)	0.6 (0.3 to 0.9)^b^	99	2.6 (1.3)	0.9 (0.6,1.2)^b^	0.3 (−0.1 to 0.7)	.14
12 mo	184	3.2 (1.3)		191	3.6 (1.2)			
Problem solving^a^								
Baseline	102	2.4 (1.3)	0.7 (0.4 to 1.0)^b^	99	2.7 (1.4)	0.6 (0.3 to 0.9)^b^	−0.1 (−0.5 to 0.3)	.72
12 mo	179	3.2 (1.4)		189	3.5 (1.4)			
Follow-up^a^								
Baseline	102	1.9 (1.1)	0.4 (0.1 to 0.7)^b^	101	2.1 (1.1)	0.6 (0.3 to 0.8)^b^	0.2 (−0.2 to 0.6)	.38
12 mo	183	2.2 (1.2)		191	2.7 (1.2)			
Perceptions of care (adapted from CAHPS)								
Had a visit with any doctors, nurses, physician assistants or other PCC in the last 6 mo, No. (%)								
Baseline	245	113 (46.1)	44 (37.1 to 50.9)^b^	240	114 (47.5)	42.9 (35.5 to 50.3)^b^	−1.1 (−10.6 to 9.1)	.79
12 mo	203	183 (90.1)		208	188 (90.4)			
Received from MCP the help needed to make changes in habits or lifestyle to improve health or prevent illness, answered yes, definitely, No. (%)								
Baseline	183	38 (20.8)	27.7 (19.5 to 36.5)^b^	180	49 (27.2)	38.4 (29.4 to 46.7)^b^	10.6 (−1.0 to 22.2)	.11
12 mo	188	91 (48.4)		192	126 (65.6)			
MCP spent enough time with patient, answered always/almost always/usually, No. (%)								
Baseline	113	57 (50.4)	21.7 (10.8 to 32.0)^b^	114	52 (45.6)	30.9 (20.4 to 42.5)^b^	9.2 (−6.5 to 24.9)	.22
12 mo	182	131 (72.0)		188	144 (76.6)			
MCP explained things in a way that was easy to understand, answered always/almost always/usually, No. (%)								
Baseline	113	61 (54.0)	23.9 (13.8 to 34.7)^b^	114	62 (54.5)	33.6 (24.3 to 42.8)^b^	9.7 (−4.8 to 23.9)	.16
12 mo	183	142 (77.6)		187	165 (87.8)			
MCP listened carefully to patient, answered always/almost always/usually, No. (%)								
Baseline	113	71 (62.8)	19.8 (9.8 to 30.0)^b^	112	69 (61.6)	21.8 (11.7 to 31.7)^b^	1.9 (−13.3 to 15.7)	.79
12 mo	182	150 (82.4)		187	156 (83.4)			
MCP showed respect for what patient had to say, answered always/almost always/usually, No. (%)								
Baseline	113	80 (70.8)	15.1 (6.0 to 24.3)^b^	113	72 (63.7)	23.5 (13.9 to 32.6)^b^	8.4 (−4.8 to 21.3)	.22
12 mo	183	157 (85.8)		187	163 (87.2)			
Rating of health care, No. (%)^i^								
Baseline	113	87 (77.0)	11.0 (2.6 to 19.3)^b^	114	87 (76.3)	16.2 (7.7 to 24.8)^b^	5.3 (−6.7 to 17.2)	.37
12 mo	183	161 (88.0)		188	174 (92.6)			
Depression, PHQ-9 Score, mean (SD)^j^								
Baseline	243	7.5 (6.1)	−2.3 (−3.2 to −1.4)^b^	231	7.1 (5.7)	−2.4 (−3.3 to −1.6)^b^	−0.2 (−1.4 to 1.1)	.79
12 mo	200	5.3 (5.1)		206	4.7 (5.0)			
Health care utilization								
Visits with geriatricians, family physicians, and internal medicine physicians, mean (SD), No. in past 6 mo								
Baseline	246	1.4 (2.0)	1.6 (1.1 to 2.1)^b^	239	1.3 (2.0)	1.7 (1.3 to 2.2)^b^	0.2 (−0.5 to 0.8)	.62
12 mo	201	3 (2.9)		205	3 (2.8)			
Visits with other types of PCC, mean (SD), No. in past 6 mo								
Baseline	246	0.2 (1.0)	0.3 (0 to 0.5)	239	0.2 (0.9)	1 (0.7 to 1.4)^b^	0.7 (0.3 to 1.2)	.001
12 mo	201	0.6 (1.6)		205	1.3 (2.3)			
Quality of life, SF-6D score, mean (SD)^k^								
Baseline	153	0.6 (0.1)	0 (0 to 0)	159	0.6 (0.09)	0 (−0.1 to 0)	0 (0 to 0)	.38
12 mo	192	0.6 (0.09)		203	0.6 (0.08)			

Abbreviations: BP, blood pressure; CAHPS, Consumer Assessment of Healthcare Providers and Systems; MCP, medical care practitioner (includes physician, nurse, and physician assistant); PACIC, Patient Assessment of Care for Chronic Conditions; PCC, primary care clinician (includes physician, nurse practitioner, and physician assistant); PHQ, patient health questionnaire; SF-6D, Short Form Six Dimension.

^a^ Scored on a scale of 1 to 5, with 1 indicating not at all and 5 indicating extremely.

^b^ The 95% CI does not include 0.

^c^ Scored on a scale of 1 to 5, with 1 being always and 5 being never.

^d^ Scored on a scale of 1 to 5, with 1 indicating never and 5 indicating all the time.

^e^ Among participants with transient ischemic attack or ischemic stroke.

^f^ Range, 10 to 40, with higher score indicating more self-efficacy.

^g^ Scored on a scale of 1 to 5, with 1 indicating strongly agree and 5 indicating strongly disagree.

^h^ Scored on a scale of 1 to 5, with 1 indicating strongly disagree and 5 indicating strongly agree.

^i^ Health care was rated on a scale of 0 (worst) to 10 (best); data here refer to patients who rated care as 6 to 10.

^j^ Scores range from 0 to 27, with higher score indicating more severe depression.

^k^ Scores range from 0 to 1, with higher score indicating better quality of life.

Subgroup analyses revealed that of 19 potential moderators, only intervention participants with severe disability and those who were working prior to the stroke had improvements in BP control compared with usual care (eTable 4 in Supplement 1). Other potential moderators, including site, race, ethnicity, and preferred language, were not associated with primary (eTable 4 in Supplement 1) or secondary outcomes. While the numerical magnitude of an effect of the intervention on BP reduction was larger at the sites where BP monitors, self-management tools, and linguistically tailored materials were not provided compared with the site where these materials were provided as part of usual care, the differences in intervention impact on BP across sites were not statistically significant (eTable 4 in Supplement 1).

In the intervention group, a total of 35 participants (14.5%) attended 3 or more clinic visits, 3 or more home visits, and 4 or more CDSMP classes; 71 participants (29.5%) attended 3 or more clinic visits, 3 or more home visits, and fewer than 4 CDSMP classes; 32 participants (13.3%) attended 2 or more clinic visits and 2 or more home visits; and 78 participants (32.4%) attended fewer than 2 clinic visits and fewer than 2 home visits; and 25 participants (10.4%) received none of the core aspects of the intervention.

**Figure 2.**
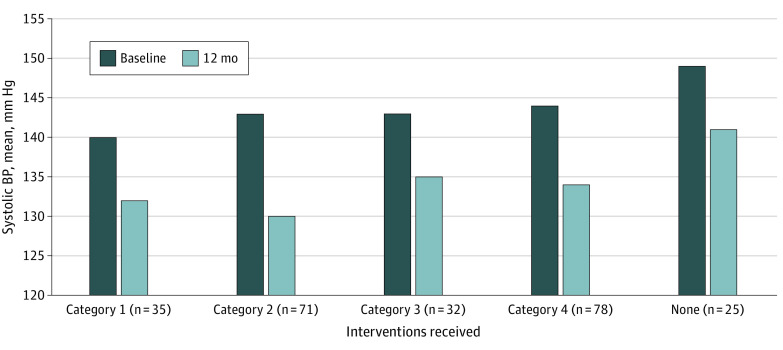
Change in Systolic Blood Pressure (BP) by Level of Participation in Intervention Individuals randomized to the intervention group were categorized by the extent of the intervention they received: (1) at least 3 clinic visits, at least 3 home visits, and at least 4 Chronic Disease Self-Management Program (CDSMP) classes; (2) at least 3 clinic visits, at least 3 home visits, and fewer than 4 CDSMP classes; (3) at least 2 clinic visits and at least 2 home visits; (4) fewer than 2 clinic visits and fewer than 2 home visits; and (5) no intervention. Changes between baseline and 12 months were not significantly different across groups.

Analysis of primary and secondary outcomes by implementation category (or dose) as a covariate did not show a dose response pattern. Figure 2 shows baseline and 12-month systolic BP by dose; changes in systolic BP did not show a dose response pattern. Multivariable analysis showed that none of the 3 core components of the intervention had a unique, significant impact on BP reduction.

Among participants with ischemic stroke, the estimated achieved RRR for recurrent stroke was modest at 15% (95% CI, 5% to 30%; *P* < .001) in the experimental group and represented one-fifth the benefit that would have been possible, given baseline risk factors and the potential impact of each risk factor intervention (modified GO score, 0.20 [95% CI, 0.06-0.38]; *P* = .009). Results did not differ from those in the usual care group (eTable 5 in Supplement 1).

## Discussion

In this randomized clinical trial of a team-based complex intervention for improving poststroke risk factor control in a racially/ethnically diverse safety-net population, the primary outcome of systolic BP improved in both intervention and control groups, without significant differences between groups. Among secondary outcomes, participants in the intervention group had larger improvements compared with usual care in self-reported salt intake and CRP level. Of hypothesized mediators, the intervention was associated with BP self-monitoring, number of visits with APCs, and statin use. Modified GO score results suggest that trial participants in both groups averted a mean of less than one-fifth of the risk of ischemic stroke they could potentially have prevented. In both study arms, despite salutary changes in BP and smoking, when considered in concert with mild worsening of non-HDL cholesterol and antithrombotic use, the relative reduction in recurrent stroke risk was likely small.

In this complex intervention, we hypothesized that home visits, clinic visits, and CDSMP workshops were core components. While 90% of participants in the intervention group received some components, only 15% of participants received the intended full dose. Numerous factors likely affected extent of participation in intervention components. We recently found that individuals with higher numbers of clinic and home visits, moderately severe disability, and later enrollment in the study (ie, after we began offering incentives and transportation) attended more CDSMP sessions, whereas those with higher chaos scores attended fewer sessions.^41^ This suggests that addressing key social determinants of health may enhance participation.

There are several potential explanations for the similar BP reduction in both groups. First, by targeting numerous vascular risk factors and using collaborative goal setting, BP control may not have been prioritized. Second, only 15% of participants in the intervention group received the full intended dose. Third, there may have been contamination of usual care, as most study clinicians also treated participants randomized to usual care. Fourth, the inability to comprehensively track the care team’s adherence and adaptations to the protocols in real time limited our ability to adjust implementation strategies in an ongoing fashion. Fifth, the site that enrolled most participants routinely offered interdisciplinary education, free BP monitors, postdischarge clinics, and some self-management tools to all patients who experienced stroke. Sixth, improvements in outcomes in usual care could have been a result of a Hawthorne effect or participants’ interactions with the outcomes team (who were culturally and linguistically concordant with the participants).

Our findings were similar to other complex interventions for poststroke vascular risk factor control in Hispanic and Black individuals in urban settings.^11,42,43^ In a 2018 randomized clinical trial by Cheng et al^11^ in the same Los Angeles safety-net setting, systolic BP decreased 17 mm Hg in the intervention group and 14 mm Hg in the usual care group, but there was no significant difference between groups. A 2020 study of Black and Hispanic survivors of hypertensive stroke randomized to usual home care vs an added 30-day nurse practitioner transitional care program vs added nurse practitioner and 60-day health coach programs similarly found a 10 mm Hg reduction in systolic BP in all groups.^42^ A randomized clinical trial by Boden-Albala et al^43^ of a skills-based, culturally tailored poststroke discharge protocol in non-Hispanic Black, non-Hispanic White, or Hispanic survivors of stroke showed no difference between individuals randomized to intervention vs usual care, although a subgroup analysis revealed a 10-mm Hg greater reduction in systolic BP in Hispanic participants vs others.

Trials of APC-CHW-physician team-based interventions in nonstroke populations have shown more impressive results. These trials differed from the SUCCEED trial by directly addressing key barriers (eg, transportation and medication cost) and having additional contact with participants. For example, a randomized clinical trial by Dennison et al^44^ of an intensive team intervention vs information and referral for hypertensive Black men found BP improvements in both groups, with more significant changes in the intervention group. Compared with the SUCCEED trial, participants received more frequent clinic visits (≥1 visit every 2-3 months) and free transportation, BP medications, and employment guidance (ie, assistance in finding employment, to increase financial stability). The Community Outreach and Cardiovascular Health (COACH) study^45^ randomized participants with cardiovascular disease to a team intervention or enhanced usual care. At 12 months, intervention participants had greater overall improvement in lipids, BP, HbA_1c_, and perceptions of care quality. In contrast to the SUCCEED trial, 70% of participants in the COACH trial had more than 4 in-person visits with the NP.

### Strengths and Limitations

This study has several strengths, including aiming to address poststroke risk factor control in a racially/ethnically diverse, high-risk population; using a theory-based behavioral intervention model; incorporating evidence-based components (eg, CCM, electronic decision support, CDSMP, and CHW home visits); adapting the intervention from lessons learned from a prior randomized clinical trial in the same setting^11^; framing the intervention with a conceptual model accounting for the mechanism of change anticipated based on literature and our prior experience^12^; community involvement in design and implementation of the intervention; allowing individual and site level variability; comprehensive intervention team training; weekly team meetings to ensure adherence to protocols and address barriers in implementation; rigorous standards for biomarker and survey collection; assessment of numerous mediators, moderators, and outcomes; and modeling the impact of multiple interventions using a recurrent stroke risk reduction tool. Many of these strengths are essential elements for developing and evaluating complex interventions.^46^

This study also has several limitations. First, the population enrolled and retained in the trial may not be representative of the entire patient population. Second, the sample size did not take into account variability in intervention delivery. Third, all CHWs were Hispanic and bilingual in English and Spanish, limiting cultural concordance with other ethnic groups. Fourth, by addressing numerous vascular risk factors and permitting tailoring, the impact of the intervention on the primary outcome may have been diluted. Fifth, variations in care delivery by clinician, site, and situation were incompletely characterized.

## Conclusions

This randomized clinical trial did not find a difference between a complex multicomponent intervention and usual care in poststroke systolic blood pressure control in participants from safety-net settings who had experienced a recent stroke. Future studies may consider characterizing why, how, and to what extent components of the SUCCEED intervention were tailored, examining which tools and strategies were preferred for addressing each of the mediators, and assessing the extent to which evidence-based protocols were followed by the care team. Future studies may consider focusing efforts on a more select suite of interventions with the strongest evidence to reduce recurrent stroke risk; determining the effectiveness of SUCCEED if fully implemented, enhanced, or simplified; using a more racially/ethnically diverse group of APCs and CHWs; and further addressing social determinants of health.

## Notes

Supplement 1. **eAppendix.** Supplementary Methods **eTable 1.** Usual Care at Study Sites **eTable 2.** Baseline Moderators **eTable 3.** Baseline Outcome Measures **eTable 4.** Changes in Systolic Blood Pressure Over Time in Usual Care vs. Intervention, by Subgroup **eTable 5.** Relative Risk Reduction of Recurrent Stroke in SUCCEED **eFigure.** SUCCEED Conceptual Model

Supplement 2. **Trial Protocol**

Supplement 3. **Data Sharing Statement**

## References

[1] Benjamin EJ, Muntner P, Alonso A, ; American Heart Association Council on Epidemiology and Prevention Statistics Committee and Stroke Statistics Subcommittee Heart disease and stroke statistics—2019 update: a report from the American Heart Association. Circulation. 2019;139(10):e56-e528. doi:10.1161/CIR.0000000000000659 30700139

[2] Hackam DG, Spence JD Combining multiple approaches for the secondary prevention of vascular events after stroke: a quantitative modeling study. Stroke. 2007;38(6):1881-1885. doi:10.1161/STROKEAHA.106.475525 17431209

[3] Cheng EM, Asch SM, Brook RH, Suboptimal control of atherosclerotic disease risk factors after cardiac and cerebrovascular procedures. Stroke. 2007;38(3):929-934. doi:10.1161/01.STR.0000257310.08310.0f 17255549

[4] Lin AM, Lin MP, Markovic D, Ovbiagele B, Sanossian N, Towfighi A Less than ideal. Stroke. 2018;50(1):A118022644. doi:10.1161/STROKEAHA.118.022644 30580724

[5] Kim O, Ovbiagele B, Valle N, Markovic D, Towfighi A Race-ethnic disparities in cardiometabolic risk profiles among stroke survivors with undiagnosed diabetes and prediabetes in the United States. J Stroke Cerebrovasc Dis. 2017;26(12):2727-2733. doi:10.1016/j.jstrokecerebrovasdis.2017.06.037 28803784

[6] Razmara A, Ovbiagele B, Markovic D, Towfighi A Patterns and predictors of blood pressure treatment, control, and outcomes among stroke survivors in the United States. J Stroke Cerebrovasc Dis. 2016;25(4):857-865. doi:10.1016/j.jstrokecerebrovasdis.2015.12.027 26778599

[7] Bodenheimer T, Wagner EH, Grumbach K Improving primary care for patients with chronic illness. JAMA. 2002;288(14):1775-1779. doi:10.1001/jama.288.14.1775 12365965

[8] Bodenheimer T, Wagner EH, Grumbach K Improving primary care for patients with chronic illness: the chronic care model, Part 2. JAMA. 2002;288(15):1909-1914. doi:10.1001/jama.288.15.1909 12377092

[9] Vickrey BG, Mittman BS, Connor KI, The effect of a disease management intervention on quality and outcomes of dementia care: a randomized, controlled trial. Ann Intern Med. 2006;145(10):713-726. doi:10.7326/0003-4819-145-10-200611210-00004 17116916

[10] Connor KI, Cheng EM, Barry F, Randomized trial of care management to improve Parkinson disease care quality. Neurology. 2019;92(16):e1831-e1842. doi:10.1212/WNL.0000000000007324 30902908 PMC6550506

[11] Cheng EM, Cunningham WE, Towfighi A, Efficacy of a chronic care-based intervention on secondary stroke prevention among vulnerable stroke survivors: a randomized controlled trial. Circ Cardiovasc Qual Outcomes. 2018;11(1):e003228. doi:10.1161/CIRCOUTCOMES.116.003228 29321134 PMC5769158

[12] Towfighi A, Cheng EM, Ayala-Rivera M, Randomized controlled trial of a coordinated care intervention to improve risk factor control after stroke or transient ischemic attack in the safety net: Secondary stroke prevention by uniting community and chronic care model teams early to end disparities (SUCCEED). BMC Neurol. 2017;17(1):24. doi:10.1186/s12883-017-0792-7 28166784 PMC5294765

[13] Rosenstock IM, Strecher VJ, Becker MH Social learning theory and the Health Belief Model. Health Educ Q. 1988;15(2):175-183. doi:10.1177/109019818801500203 3378902

[14] Chobanian AV, Bakris GL, Black HR, ; Joint National Committee on Prevention, Detection, Evaluation, and Treatment of High Blood Pressure. National Heart, Lung, and Blood Institute; National High Blood Pressure Education Program Coordinating Committee Seventh report of the Joint National Committee on Prevention, Detection, Evaluation, and Treatment of High Blood Pressure. Hypertension. 2003;42(6):1206-1252. doi:10.1161/01.HYP.0000107251.49515.c2 14656957

[15] Boan AD, Lackland DT, Ovbiagele B Lowering of blood pressure for recurrent stroke prevention. Stroke. 2014;45(8):2506-2513. doi:10.1161/STROKEAHA.114.003666 24984744 PMC4134881

[16] Ovbiagele B Low-normal systolic blood pressure and secondary stroke risk. J Stroke Cerebrovasc Dis. 2013;22(5):633-638. doi:10.1016/j.jstrokecerebrovasdis.2011.12.003 22244715

[17] Ovbiagele B, Diener HC, Yusuf S, ; PROFESS Investigators Level of systolic blood pressure within the normal range and risk of recurrent stroke. JAMA. 2011;306(19):2137-2144. doi:10.1001/jama.2011.1650 22089721

[18] Lin MP, Ovbiagele B, Markovic D, Towfighi A Systolic blood pressure and mortality after stroke: too low, no go? Stroke. 2015;46(5):1307-1313. doi:10.1161/STROKEAHA.115.008821 25765723

[19] Lorig KR, Ritter P, Stewart AL, Chronic disease self-management program: 2-year health status and health care utilization outcomes. Med Care. 2001;39(11):1217-1223. doi:10.1097/00005650-200111000-00008 11606875

[20] Ramirez M, Wu S, Ryan G, Towfighi A, Vickrey BG Using beta-version mHealth technology for team-based care management to support stroke prevention: an assessment of utility and challenges. JMIR Res Protoc. 2017;6(5):e94. doi:10.2196/resprot.7106 28536094 PMC5461415

[21] Craig CL, Marshall AL, Sjöström M, International physical activity questionnaire: 12-country reliability and validity. Med Sci Sports Exerc. 2003;35(8):1381-1395. doi:10.1249/01.MSS.0000078924.61453.FB 12900694

[22] UCLA Center for Health Policy Research California health interview survey 2011-2012. Accessed January 4, 2021. http://healthpolicy.ucla.edu/chis/design/Pages/questionnairesEnglish.aspx

[23] Centers for Disease Control and Prevention 2013 Behavioral Risk Factor Surveillance System Survey Questionnaire. Revised December 28, 2012 Accessed January 4, 2021. https://www.cdc.gov/brfss/questionnaires/pdf-ques/2013-BRFSS_English.pdf

[24] Haun J, Luther S, Dodd V, Donaldson P Measurement variation across health literacy assessments: implications for assessment selection in research and practice. J Health Commun. 2012;17(suppl 3):141-159. doi:10.1080/10810730.2012.712615 23030567

[25] Powers BJ, Oddone EZ, Grubber JM, Olsen MK, Bosworth HB Perceived and actual stroke risk among men with hypertension. J Clin Hypertens (Greenwich). 2008;10(4):287-294. doi:10.1111/j.1751-7176.2008.07797.x 18401226 PMC8110066

[26] Nicol MB, Thrift AG Knowledge of risk factors and warning signs of stroke. Vasc Health Risk Manag. 2005;1(2):137-147. doi:10.2147/vhrm.1.2.137.64085 17315400 PMC1993942

[27] Schneider AT, Pancioli AM, Khoury JC, Trends in community knowledge of the warning signs and risk factors for stroke. JAMA. 2003;289(3):343-346. doi:10.1001/jama.289.3.343 12525235

[28] Chesney MA, Ickovics JR, Chambers DB, ; Patient Care Committee & Adherence Working Group of the Outcomes Committee of the Adult AIDS Clinical Trials Group (AACTG) Self-reported adherence to antiretroviral medications among participants in HIV clinical trials: the AACTG adherence instruments. AIDS Care. 2000;12(3):255-266. doi:10.1080/09540120050042891 10928201

[29] Simoni JM, Kurth AE, Pearson CR, Pantalone DW, Merrill JO, Frick PA Self-report measures of antiretroviral therapy adherence: a review with recommendations for HIV research and clinical management. AIDS Behav. 2006;10(3):227-245. doi:10.1007/s10461-006-9078-6 16783535 PMC4083461

[30] Luszczynska A, Scholz U, Schwarzer R The general self-efficacy scale: multicultural validation studies. J Psychol. 2005;139(5):439-457. doi:10.3200/JRLP.139.5.439-457 16285214

[31] Cunningham WE, Andersen RM, Katz MH, The impact of competing subsistence needs and barriers on access to medical care for persons with human immunodeficiency virus receiving care in the United States. Med Care. 1999;37(12):1270-1281. doi:10.1097/00005650-199912000-00010 10599608

[32] Agency for Healthcare Research and Quality CAHPS Clinician & Group Survey. Revised March 2019 Accessed January 4, 2021. https://www.ahrq.gov/cahps/surveys-guidance/cg/index.html

[33] Glasgow RE, Wagner EH, Schaefer J, Mahoney LD, Reid RJ, Greene SM Development and validation of the Patient Assessment of Chronic Illness Care (PACIC). Med Care. 2005;43(5):436-444. doi:10.1097/01.mlr.0000160375.47920.8c 15838407

[34] Kroenke K, Spitzer RL, Williams JB The PHQ-9: validity of a brief depression severity measure. J Gen Intern Med. 2001;16(9):606-613. doi:10.1046/j.1525-1497.2001.016009606.x 11556941 PMC1495268

[35] RAND Corporation HCSUS baseline questionnaire. Accessed April 19, 2020. https://www.rand.org/health-care/projects/hcsus/Base.html

[36] Wong ST, Nordstokke D, Gregorich S, Pérez-Stable EJ Measurement of social support across women from four ethnic groups: evidence of factorial invariance. J Cross Cult Gerontol. 2010;25(1):45-58. doi:10.1007/s10823-010-9111-0 20182911 PMC2836242

[37] Kharroubi SA, Brazier JE, Roberts J, O’Hagan A Modelling SF-6D health state preference data using a nonparametric Bayesian method. J Health Econ. 2007;26(3):597-612. doi:10.1016/j.jhealeco.2006.09.002 17069909

[38] Wong MD, Sarkisian CA, Davis C, Kinsler J, Cunningham WE The association between life chaos, health care use, and health status among HIV-infected persons. J Gen Intern Med. 2007;22(9):1286-1291. doi:10.1007/s11606-007-0265-6 17597350 PMC2219764

[39] Lee S, O’Neill AH, Ihara ES, Chae DH Change in self-reported health status among immigrants in the United States: associations with measures of acculturation. PLoS One. 2013;8(10):e76494. doi:10.1371/journal.pone.0076494 24098515 PMC3788132

[40] Richards A, Jackson NJ, Cheng EM, Derivation and application of a tool to estimate benefits from multiple therapies that reduce recurrent stroke risk. Stroke. 2020;51(5):1563-1569. doi:10.1161/STROKEAHA.119.027160 32200759 PMC7185059

[41] Lin AM, Vickrey BG, Barry F, Factors associated with participation in the chronic disease self-management program: findings from the SUCCEED Trial. Stroke. 2020;51(10):2910-2917. doi:10.1161/STROKEAHA.119.028022 32912091 PMC8269960

[42] Feldman PH, McDonald MV, Trachtenberg M, Reducing hypertension in a poststroke Black and Hispanic home care population: results of a pragmatic randomized controlled trial. Am J Hypertens. 2020;33(4):362-370. doi:10.1093/ajh/hpz148 31541606 PMC7109355

[43] Boden-Albala B, Goldmann E, Parikh NS, Efficacy of a discharge educational strategy vs standard discharge care on reduction of vascular risk in patients with stroke and transient ischemic attack: the DESERVE randomized clinical trial. JAMA Neurol. 2019;76(1):20-27. doi:10.1001/jamaneurol.2018.2926 30304326 PMC6439868

[44] Dennison CR, Post WS, Kim MT, Underserved urban African American men: hypertension trial outcomes and mortality during 5 years. Am J Hypertens. 2007;20(2):164-171. doi:10.1016/j.amjhyper.2006.08.003 17261462

[45] Allen JK, Dennison-Himmelfarb CR, Szanton SL, Community Outreach and Cardiovascular Health (COACH) Trial: a randomized, controlled trial of nurse practitioner/community health worker cardiovascular disease risk reduction in urban community health centers. Circ Cardiovasc Qual Outcomes. 2011;4(6):595-602. doi:10.1161/CIRCOUTCOMES.111.961573 21953407 PMC3218795

[46] Craig P, Dieppe P, Macintyre S, Michie S, Nazareth I, Petticrew M; Medical Research Council Guidance Developing and evaluating complex interventions: the new Medical Research Council guidance. BMJ. 2008;337:a1655. doi:10.1136/bmj.a1655 18824488 PMC2769032

[47] Lorig K, Holman H, Sobel D, Living a Healthy Life With Chronic Conditions. 4th ed Bull Publishing; 2012.

[48] Gonzalez V, Hernandez-Marin M, Lorig K, Tomando Control de su Salud. 4th ed Bull Publishing; 2013.

