# Supplementary material for: Effect of a Coordinated Community and Chronic Care Model Team Intervention vs Usual Care on Systolic Blood Pressure in Patients With Stroke or Transient Ischemic Attack: The SUCCEED Randomized Clinical Trial
Source: JAMA Netw Open. 2021 Feb 15;4(2):e2036227. doi: 10.1001/jamanetworkopen.2020.36227 (PMC7885035; doi:10.1001/jamanetworkopen.2020.36227)
Supplement: Supplement 1. — eAppendix. Supplementary Methods eTable 1. Usual Care at Study Sites eTable 2. Baseline Moderators eTable 3. Baseline Outcome Measures eTable 4. Changes in Systolic Blood Pressure Over Time in Usual Care vs. Intervention, by Subgroup eTable 5. Relative Risk Reduction of Recurrent Stroke in SUCCEED eFigure. SUCCEED Conceptual Model [file jamanetwopen-e2036227-s001.pdf]

Supplemental Online Content

Towfighi A, Cheng EM, Ayala-Rivera M, et al; Secondary Stroke Prevention by Uniting Community and Chronic Care Model Teams Early to End Disparities (SUCCEED) Investigators. Effect of a coordinated community and chronic care model team intervention vs usual care on systolic blood pressure in patients with stroke or transient ischemic attack: the SUCCEED randomized clinical trial. *JAMA Netw Open*. 2021;4(2):e2036227. doi:10.1001/jamanetworkopen.2020.36227

eAppendix. Supplementary Methods
eTable 1. Usual Care at Study Sites
eTable 2. Baseline Moderators
eTable 3. Baseline Outcome Measures
eTable 4. Changes in Systolic Blood Pressure Over Time in Usual Care vs. Intervention, by Subgroup
eTable 5. Relative Risk Reduction of Recurrent Stroke in SUCCEED
eFigure. SUCCEED Conceptual Model

This supplemental material has been provided by the authors to give readers additional information about their work.

© 2021 Towfighi A et al. *JAMA Network Open.*

eAppendix. Supplementary Methods

Usual Care at Study Sites

Stroke specialists in the LAC-DHS safety-net system collaboratively developed expected practices for secondary stroke prevention for primary care providers, outlining evidence-based management of patients with prior stroke (Table 1). The expected practices were available on the intranet and eConsult site for all primary care providers throughout LAC-DHS and at community partner sites. The site the enrolled the majority of participants (Rancho Los Amigos National Rehabilitation Center) provided an educational binder (developed by an interdisciplinary team including feedback by patients and caregivers) and BP monitor to all patients prior to discharge from the hospital. Providers taught patients how to check, log and track their BP and scheduled patients in an APP-staffed Neurology Transition Clinic within 2 weeks of discharge from the hospital. The four other sites provided either a folder with resources and American Heart Association handouts or handouts printed from the electronic medical record and follow up with primary care and neurology.

Sensitivity Analyses

The first sensitivity model excluded 12 subjects who were enrolled at the beginning of the study who, at baseline, had SBP< 130 mm Hg, did not have a history of hypertension, and were not taking hypertensive medications. Two additional sensitivity analyses (one for the main analysis and the other for the first sensitivity analysis) accounted for missing data due to non-collection of the 12-month survey by including predicted probabilities as a covariate, where the predicted probabilities were derived from logistic regression models with whether or not the 12-month data were missing as the dependent variable, and covariates of age, survey language, and marital status. The final sensitivity analysis replaced the categorical measure of time (survey) with a continuous measure of time, defined as the number of days from the baseline survey. A 5% significance level was used throughout and two-sided tests were used where appropriate.

Modified Global Outcome (GO) Score

The modified Global Outcome Score (GO Score) is a measure of the proportion of potential benefit (RRR)
possible with ideal implementation of all evidence-based interventions for ischemic stroke. It is calculated as the ratio of the estimated RRR achieved at the end of the trial divided by the estimated ideal RRR when all risk factors are optimally treated for each individual: $RRR_{achieved}/RRR_{ideal}$. Ideal relative risk reduction (RRR_{ideal}) represents the expected reduction in risk of recurrent ischemic stroke that would be possible if all trial participants with ischemic stroke received and adhered to all five evidence-based interventions optimally: blood pressure and lipid lowering, smoking cessation, and adherence to warfarin or antiplatelet therapy (for participants with and without presumed atrial fibrillation, respectively). $RRR_{achieved}$ is the relative risk reduction associated with changes in those five factors observed in the trial. Relative risk reduction estimates were calculated for participants with ischemic stroke and complete data on blood pressure and lipids at 12 months (n=317).

© 2021 Towfighi A et al. *JAMA Network Open.*

eTable 1. Usual Care at Study Sites

**Component**	**Rancho Los Amigos National Rehabilitation Center** (n=325)	**Harbor-UCLA Medical Center** (n=74)	**LAC+USC Medical Center** (n=67)	**Olive View-UCLA Medical Center** (n=6)	**Cedars-Sinai Medical Center** (n=15)
Education Materials	Education binder with linguistically tailored materials, self-management tools, local resources	Education handouts from electronic medical record	Education handouts from electronic medical record	Education handouts from electronic medical record	Education folder with AHA materials, magnet, bookmark, and stroke support group listings
Free Blood Pressure Monitor	X				
Expected Practice for Secondary Stroke Prevention	X	X	X	X	
Clinic Appointment with Neurology APP within 2 Weeks of Discharge	X				
Appointment with Neurologist	Select patients, depending on complexity	X	X	X	X

APP: advanced practice provider

© 2021 Towfighi A et al. *JAMA Network Open.*

eTable 2. Baseline Moderators

Characteristic	Total (N=487)	Usual Care (N=246)	Intervention (N=241)	p-value
**Chaos Score (scale 6 [least chaos]- 30 [most chaos]), mean (SD)**	16.23 (5.2)	16.4 (5.4)	16.05 (5.1)	0.47
**Competing Needs, n (%)**				
Went without food, clothing, housing, etc. because needed money for health care	35 (7.3)	16 (6.6)	19 (8.1)	0.60
Went without needed health care because needed money for food, clothing, housing, etc	108 (22.6)	51 (21.2)	57 (24.2)	0.44
Unable to fill or could not afford a prescription, could not buy needed medical supplies, or could not pay medical bills	109 (23.4)	51 (21.6)	58 (25.2)	0.36
Unable to pay rent or mortgage	125 (26.8)	63 (26.9)	62 (26.6)	0.94
Put off going to the doctor, clinic or hospital for medical care because could not get off work	99 (24.9)	49 (23.6)	50 (26.5)	0.50
Put off going to the doctor, clinic or hospital for medical care because too sick	58 (12.4)	34 (14.3)	24 (10.4)	0.19
Put off going to the doctor, clinic or hospital for medical care because didn't have a way to get there	70 (15.0)	34 (14.5)	36 (15.6)	0.74
Put off going to the doctor, clinic or hospital for medical care because taking care of someone else was more important	62 (13.2)	26 (11.0)	36 (15.5)	0.15
**Acculturation, n (%)**				
How often do you speak in English in your home?				0.36
Almost never	192 (39.7)	102 (41.6)	90 (37.7)	
Sometimes	79 (16.3)	34 (13.9)	45 (18.8)	
Often	30 (6.2)	13 (5.3)	17 (7.1)	
Almost always	183 (37.8)	96 (39.2)	87 (36.4)	
How often do you speak English outside of your home when you are with friends?				0.86
Almost never	147 (30.4)	74 (30.3)	73 (30.4)	

© 2021 Towfighi A et al. *JAMA Network Open.*

Sometimes	109 (22.5)	56 (23.0)	53 (22.1)	
Often	26 (5.4)	11 (4.5)	15 (6.3)	
Almost always	202 (41.7)	103 (42.2)	99 (41.3)	
How well would you say you understand English when someone is speaking to you?				0.37
Very well	198 (40.8)	104 (42.5)	94 (39.2)	
Well	70 (14.4)	35 (14.3)	35 (14.6)	
Not well	157 (32.4)	82 (33.5)	75 (31.3)	
Not at all	60 (12.4)	24 (9.8)	36 (15.0)	
How well would you say you speak English?				0.81
Very well	169 (34.9)	86 (35.1)	83 (34.7)	
Well	77 (15.9)	37 (15.1)	40 (16.7)	
Not well	160 (33.1)	85 (34.7)	75 (31.4)	
Not at all	78 (16.1)	37 (15.1)	41 (17.2)	

© 2021 Towfighi A et al. *JAMA Network Open.*

eTable 3. Baseline Outcome Measures

**Baseline Variables**	**Usual Care (N=246)**		**Intervention (N=241)**		**p-value**
**Primary**					
SBP ≤ 130 mmHg, n (%)	246	53 (21.5)	241	62 (25.7)	0.28
SBP, mm Hg, mean (SD)	246	145.7 (18.6)	241	143.2 (17.1)	0.12
**Secondary**					
Non-HDL, mg/dL, mean (SD)	201	95.7 (40.4)	191	94.1 (42.3)	0.69
HbA1c, %, mean (SD)	160	6.8 (1.4)	161	6.8 (1.6)	0.92
CRP (log), mean (SD)	198	0.6 (1.2)	202	1 (1.2)	**<0.001**
BMI kg/m^2, mean (SD)	173	29.1 (5.1)	163	29.2 (5.7)	0.80
Waist circumference, cm, mean (SD)	100	103.1 (15.8)	73	100.7 (16.0)	0.32
Physical activity, IPAQ MET minutes, per week, median (IQR)	244	540 (0, 2040)	239	600 (40, 2400)	0.44
≥ 5 daily servings of fruit/vegetables, n (%)	244	30 (12.3)	239	20 (8.4)	0.18
0 daily servings of soda, n (%)	245	69 (28.2)	240	69 (28.8)	0.92
Reducing/watching salt intake, n (%)	245	134 (54.7)	240	104 (43.3)	**0.01**
Not smoking, n (%)	243	192 (79.0)	240	183 (76.3)	0.51
Taking antithrombotic (for participants with ischemic stroke or TIA), n (%)	206	205 (99.5)	199	197 (99.0)	0.62

SBP: systolic blood pressure; HDL: high density lipoprotein cholesterol; HbA1c: hemoglobin A1c; CRP: C reactive protein; BMI: body mass index; IPAQ: International Physical Activity Questionnaire; TIA: transient ischemic attack

© 2021 Towfighi A et al. *JAMA Network Open.*

eTable 4. Changes in Systolic Blood Pressure Over Time in Usual Care vs. Intervention, by Subgroup

	Usual Care						Intervention						
	Baseline		3 Month		12 Month		Baseline		3 Month		12 Month		P value
	n	SBP, mmHg, Mean (SD)	n	SBP, mmHg, Mean (SD)	n	SBP, mmHg, Mean (SD)	n	SBP, mmHg, Mean (SD)	n	SBP, mmHg, Mean (SD)	n	SBP, mmHg, Mean (SD)	
**Sex**													
Male	154	146 (18)	136	136 (19)	130	138 (22)	163	143 (17)	143	134 (21)	138	132 (20)	0.42
Female	92	145 (19)	81	136 (22)	74	136 (22)	78	143 (18)	67	134 (20)	68	136 (22)	0.91
**Country of Birth**													
US	69	139 (14)	53	134 (16)	53	137 (20)	65	141 (16)	55	133 (24)	51	136 (26)	0.61
Not US	176	148 (20)	163	137 (21)	151	137 (23)	176	144 (18)	155	134 (19)	155	132 (18)	0.77
**Language of Survey**													
English	106	142 (16)	87	133 (17)	82	136 (22)	101	142 (16)	85	134 (24)	81	133 (23)	0.30
Spanish	140	149 (20)	130	138 (22)	122	138 (22)	140	144 (18)	125	134 (18)	125	133 (19)	0.96
**Ethnicity**													
Hispanic	177	147 (19)	163	137 (21)	154	136 (21)	170	145 (17)	151	134 (20)	150	133 (19)	0.99
Not Hispanic	69	142 (16)	54	134 (17)	50	139 (24)	71	140 (16)	59	134 (23)	56	132 (23)	0.22
**Site**													
Harbor	37	146 (19)	34	132 (18)	33	135 (18)	37	144 (17)	33	131 (19)	30	126 (22)	0.26
USC	34	156 (20)	29	149 (29)	26	146 (27)	33	151 (23)	29	139 (22)	29	137 (25)	0.87
Rancho	162	143 (18)	144	135 (18)	136	135 (22)	163	141 (15)	141	134 (21)	142	133 (18)	0.92
**Type of Stroke**													
Ischemic/TIA	208	146 (19)	184	136 (21)	171	138 (22)	198	144 (17)	170	134 (20)	168	133 (21)	0.70

© 2021 Towfighi A et al. *JAMA Network Open.*

Intracerebral Hemorrhage	38	143 (15)	33	134 (12)	33	133 (20)	43	142 (16)	40	133 (21)	38	132 (20)	0.99
**Education**													
Some college	73	145 (18)	67	131 (16)	59	134 (23)	74	139 (15)	62	130 (22)	58	128 (22)	0.24
High school graduate or equivalent	22	140 (12)	18	130 (18)	17	143 (23)	15	152 (17)	15	145 (22)	15	131 (14)	**0.001**
Some high school	56	146 (18)	48	138 (20)	52	140 (23)	59	143 (16)	50	134 (22)	49	138 (24)	0.75
8th grade or less	93	148 (21)	82	140 (23)	75	136 (20)	90	146 (19)	80	135 (18)	81	134 (17)	0.53
**Working for pay, part- or full-time prior to stroke**													
Yes	141	147 (19)	127	134 (18)	117	138 (22)	125	145 (19)	112	136 (22)	108	132 (19)	**0.02**
No	102	144 (18)	87	139 (23)	85	135 (22)	115	141 (15)	97	131 (19)	97	135 (22)	0.09
**Marital Status**													
Married / Domestic partnership	114	146 (21)	105	134 (21)	101	136 (21)	105	143 (16)	96	132 (19)	94	130 (18)	0.47
Other	131	145 (17)	111	138 (20)	103	138 (23)	136	144 (18)	114	135 (22)	112	136 (22)	0.94
**Insurance**													
Government	157	145 (17)	138	136 (19)	136	137 (21)	151	141 (16)	131	131 (20)	131	131 (20)	0.78

© 2021 Towfighi A et al. *JAMA Network Open.*

Private Insurance	21	144 (16)	19	130 (15)	17	139 (14)	24	145 (18)	21	144 (16)	21	146 (21)	0.21
No health insurance	45	149 (23)	38	140 (26)	33	133 (25)	48	149 (20)	42	134 (21)	40	130 (18)	0.61
**Primary Care Provider**													
Yes	120	143 (18)	109	136 (21)	103	134 (20)	111	142 (16)	95	132 (20)	97	133 (21)	0.47
No	123	148 (19)	105	135 (20)	99	139 (23)	130	144 (18)	115	136 (21)	109	133 (20)	0.16
**Modified Rankin Scale**													
No disability (0)	33	149 (19)	32	139 (21)	31	138 (19)	33	146 (19)	28	134 (26)	28	133 (25)	0.91
Not significant (1)	49	146 (17)	42	137 (21)	38	137 (22)	32	144 (18)	28	137 (18)	27	139 (22)	0.71
Slight (2)	31	152 (20)	26	143 (24)	26	142 (23)	41	144 (20)	37	135 (16)	36	133 (20)	0.91
Moderate (3)	64	147 (21)	57	136 (21)	54	134 (20)	73	140 (15)	64	133 (19)	66	134 (19)	0.28
Moderate/Severe (4)	51	139 (13)	44	131 (16)	40	137 (27)	50	142 (14)	42	129 (22)	40	127 (18)	**0.04**
**NIH Stroke Scale**													
Mild (≤ 5)	145	148 (20)	129	137 (21)	118	137 (21)	136	144 (18)	119	136 (21.)	118	133 (21)	0.66
Moderate/Severe (>5)	86	142 (17)	73	132 (16)	72	136 (24)	94	142 (16)	81	131 (20)	79	131 (20)	0.46
**Race**													
Asian	15	141 (14)	14	126 (18)	10	131 (22)	15	141 (20)	12	130 (23)	13	128 (15)	0.68
Black	42	142 (14)	29	135 (15)	33	136 (22)	45	141 (15)	39	137 (24)	34	136 (28)	0.94
White	167	148 (19)	153	139 (21)	143	137 (23)	168	144 (18)	148	133 (19)	148	133 (19)	0.64
Other	15	142 (24)	14	122 (10)	12	138 (20)	9	141 (17)	7	139 (19)	7	140 (20)	0.14
**Living Situation**													

© 2021 Towfighi A et al. *JAMA Network Open.*

Own home	167	147 (20)	147	134 (19)	137	136 (22)	171	143 (17)	150	134 (21)	150	133 (21)	0.28
With a relative	46	146 (17)	38	137 (21)	39	136 (19)	47	145 (19)	40	132 (21)	41	132 (17)	0.61
Other	21	142 (15)	21	146 (27)	18	138 (26)	18	140 (13)	16	129 (16)	12	134 (25)	0.19
**# of Comorbidities**													
None	57	148 (21)	46	134 (19)	45	137 (23)	48	142 (16)	44	134 (23)	41	130 (16)	0.33
≥1	189	145 (18)	171	137 (21)	159	137 (22)	193	144 (17)	166	134 (20)	165	134 (21)	0.78
**Competing Needs**													
During the 6 months prior to your stroke or TIA, did you ever have to go without health care that you needed because you needed the money for food, clothing, housing, etc?													
Yes	51	149 (22)	45	132 (21)	41	143 (22)	57	143 (18)	51	134 (21)	47	133 (19)	0.06
No	190	144 (17)	167	137 (20)	158	135 (21)	179	143 (17)	154	134 (20)	154	133 (21)	0.76
During the 6 months													

© 2021 Towfighi A et al. *JAMA Network Open.*

prior to your stroke or TIA, were there times when you were unable to fill or could not afford a prescription, could not buy needed medical supplies, or could not pay your medical bills?													
Yes	51	149 (20)	46	136 (24)	46	142 (23)	58	144 (18)	53	133 (17)	49	133 (16)	0.35
No	185	144 (17)	163	136 (20)	150	135 (21)	172	143 (17)	146	134 (22)	146	133 (22)	0.97
During the 6 months prior to your stroke or TIA, were there times when you were unable to fill or could not afford a prescription, could not													

© 2021 Towfighi A et al. *JAMA Network Open.*

buy needed medical supplies, or could not pay your medical bills?													
Yes	63	148 (19)	57	138 (22)	54	136 (21)	62	148 (17.)	59	136 (19)	55	131 (19)	0.55
No	171	144 (17)	150	134 (18)	141	136 (21)	171	141 (17)	145	133 (21)	143	134 (21)	0.88
During the 6 months prior to your stroke or TIA, did you ever put off going to the doctor, clinic or hospital for medical care (for 4 reasons)													
Yes	81	148 (21)	74	132 (17)	67	138 (21)	71	146 (19)	65	133 (21)	64	130 (20)	0.07
No	126	142 (15)	109	136 (19)	106	135 (21)	114	142 (17)	99	135 (20)	97	133 (20)	0.78
**Baseline SBP, mm Hg**													
< 140	124	132 (5)	107	131 (16)	106	131 (19)	126	130 (6)	110	129 (19)	110	130 (19)	0.98
< 160	72	149 (6)	66	139 (20)	61	138 (22)	80	150 (6)	71	138 (21)	68	133 (17)	0.26
≥ 160	50	176 (13)	44	145 (25)	37	150 (24)	35	174 (13)	29	141 (20)	28	144 (29)	0.80
**Baseline SBP, mm Hg**													
< 140	124	132 (5)	107	131 (16)	106	131 (19)	126	130 (6)	110	129 (19)	110	130 (19)	0.98

© 2021 Towfighi A et al. *JAMA Network Open.*

≥ 140	122	160 (16)	110	142 (22)	98	143 (23)	115	157 (14)	100	139 (20)	96	136 (22)	0.45
**Age, years**													
< 55	102	145 (18)	89	132 (18)	87	136 (21)	97	143 (19)	86	131 (22)	88	133 (22)	0.84
55 - 65	96	145 (18)	90	137 (21)	82	137 (24)	97	141 (15)	87	135 (18)	83	134 (19)	0.79
≥65	48	150 (20)	38	143 (23)	35	138 (19)	47	148 (16)	37	138 (20)	35	133 (20)	0.79
**Chaos**													
≤18	148	145 (18)	134	135 (19)	122	137 (22)	162	143 (18)	141	133 (21)	140	132 (20)	0.65
>18	96	146 (19)	81	137 (22)	80	137 (22)	72	144 (16)	63	134 (21)	60	134 (21)	0.85
**Days from 1st randomization**													
≤ Q 1 (347)	62	146 (20)	51	137 (17)	52	131 (22)	60	145 (20)	49	134 (17)	58	129 (20)	0.94
≤ Q 2 (712)	64	144 (17)	59	135 (23)	52	140 (18)	60	143 (14)	56	138 (21)	52	137 (19)	0.43
≤ Q 3 (942)	60	149 (19)	55	137 (20)	53	136 (22)	60	144 (19)	48	135 (22)	44	133 (19)	0.76
> Q 3	60	145 (18)	52	136 (21)	47	141 (26)	61	141 (14)	57	129 (21)	52	133 (23)	0.36
**Recruitment Location**													
Inpatient - Acute	183	144 (18)	160	136 (21)	152	138 (23)	192	142 (17)	167	134 (20)	165	132 (20)	0.25
Inpatient - Rehabilitation	45	153 (22)	41	134 (20)	38	134 (21)	39	150 (18)	34	135 (22)	34	138 (21)	0.43
Outpatient	18	145 (13)	16	140 (16)	14	138 (16)	10	146 (20)	9	132 (19)	7	143 (25)	0.18
**Acculturation**													
How often do you speak English in your home?													
Almost never	102	152 (20)	94	139 (23)	85	138 (20)	90	145 (19)	78	133 (17)	78	132 (19)	0.89

© 2021 Towfighi A et al. *JAMA Network Open.*

Sometimes	34	138 (13)	30	137 (20)	30	139 (23)	45	144 (17)	39	136 (21)	40	135 (19)	0.20
Often	13	142 (19)	13	136 (13)	12	124 (21)	17	144 (19)	14	136 (19)	16	131 (18)	0.49
Almost Always	96	142 (16)	79	132 (17)	76	137 (23)	87	141 (15)	77	133 (23)	70	133 (23)	0.27
How often do you speak English outside of your home when with friends?													
Almost never	74	150 (20)	66	139 (25)	59	138 (22)	73	142 (17)	64	134 (19)	64	134 (22)	0.70
Sometimes	56	146 (19)	54	137 (19)	48	136 (23)	53	148 (20)	48	131 (19)	47	132 (14)	0.20
Often	11	146 (21)	11	143 (19)	10	134 (21)	15	139 (16)	14	137 (24)	13	135 (16)	0.45
Almost Always	103	142 (16)	84	133 (17)	85	137 (22)	99	142 (16)	83	135 (22)	81	132 (23)	0.09
How well would you say you understand English when someone is speaking to you?													
Very well	104	144 (17)	87	133 (17)	82	138 (22)	94	142 (15)	82	135 (24)	77	134 (23)	0.22
Well	35	144 (17)	32	131 (16)	30	134 (21)	35	144 (18)	30	130 (14)	32	130 (16)	0.76

© 2021 Towfighi A et al. *JAMA Network Open.*

Not well	82	147 (19)	77	139 (23)	74	136 (22)	75	146 (19)	67	134 (20)	65	133 (19)	0.66
Not at all	24	154 (23)	20	146 (27)	18	141 (23)	36	141 (18)	30	134 (18)	31	134 (22)	0.77
How well would you say you speak English?													
Very well	86	142 (16)	70	132 (17)	67	138 (23)	83	142 (15)	73	132 (24)	66	132 (24)	0.12
Well	37	146 (20)	33	138 (15)	30	132 (19)	40	141 (16)	33	138 (19)	35	133 (14)	0.35
Not well	85	148 (18)	80	137 (23)	77	136 (22)	75	145 (18)	67	133 (18)	67	132 (18)	0.87
Not at all	37	149 (22)	33	141 (25)	29	140 (22)	41	146 (20)	35	135 (17)	36	136 (24)	0.86

SBP: systolic blood pressure

© 2021 Towfighi A et al. *JAMA Network Open.*

eTable 5. Relative Risk Reduction of Recurrent Stroke in SUCCEED

**Outcome**	**Usual Care Change from Baseline**	**Intervention Change from Baseline**	**Differences in changes from baseline between usual care and intervention (95% CI)**
Ideal Relative Risk Reduction (RRR_{ideal}), ratio (95% CI)	0.79 (0.78, 0.80)	0.78 (0.77, 0.80)	-0.01 (-0.03, 0.01)
Achieved Relative Risk Reduction ($RRR_{acheived}$), ratio (95% CI)	0.09 (-0.05, 0.22)	0.15 (0.05, 0.30)	0.06 (-0.10, 0.28)
Modified GO Score ($RRR_{acheived}/RRR_{ideal}$), ratio (95% CI)	0.12 (-0.06, 0.28)	0.20 (0.06, 0.38)	0.08 (-0.12, 0.35)

RRR_{ideal}: ideal relative risk reduction in risk of recurrent stroke - the expected reduction in risk of recurrent ischemic stroke that would be possible if all trial participants with ischemic stroke received and adhered to all five evidence-based interventions optimally (blood pressure and lipid lowering; smoking cessation; and adherence to warfarin or antiplatelet therapy, for participants with and without presumed atrial fibrillation, respectively)

$RRR_{achieved}$: the relative risk reduction of recurrent stroke associated with changes in above five factors observed in the trial

GO Score: Global Outcome Score - represents the proportion of potentially preventable stroke risk reduction achieved with the level of care provided at the end of the trial, given the level of care received at the beginning of the trial

© 2021 Towfighi A et al. *JAMA Network Open.*

eFigure. SUCCEED Conceptual Model

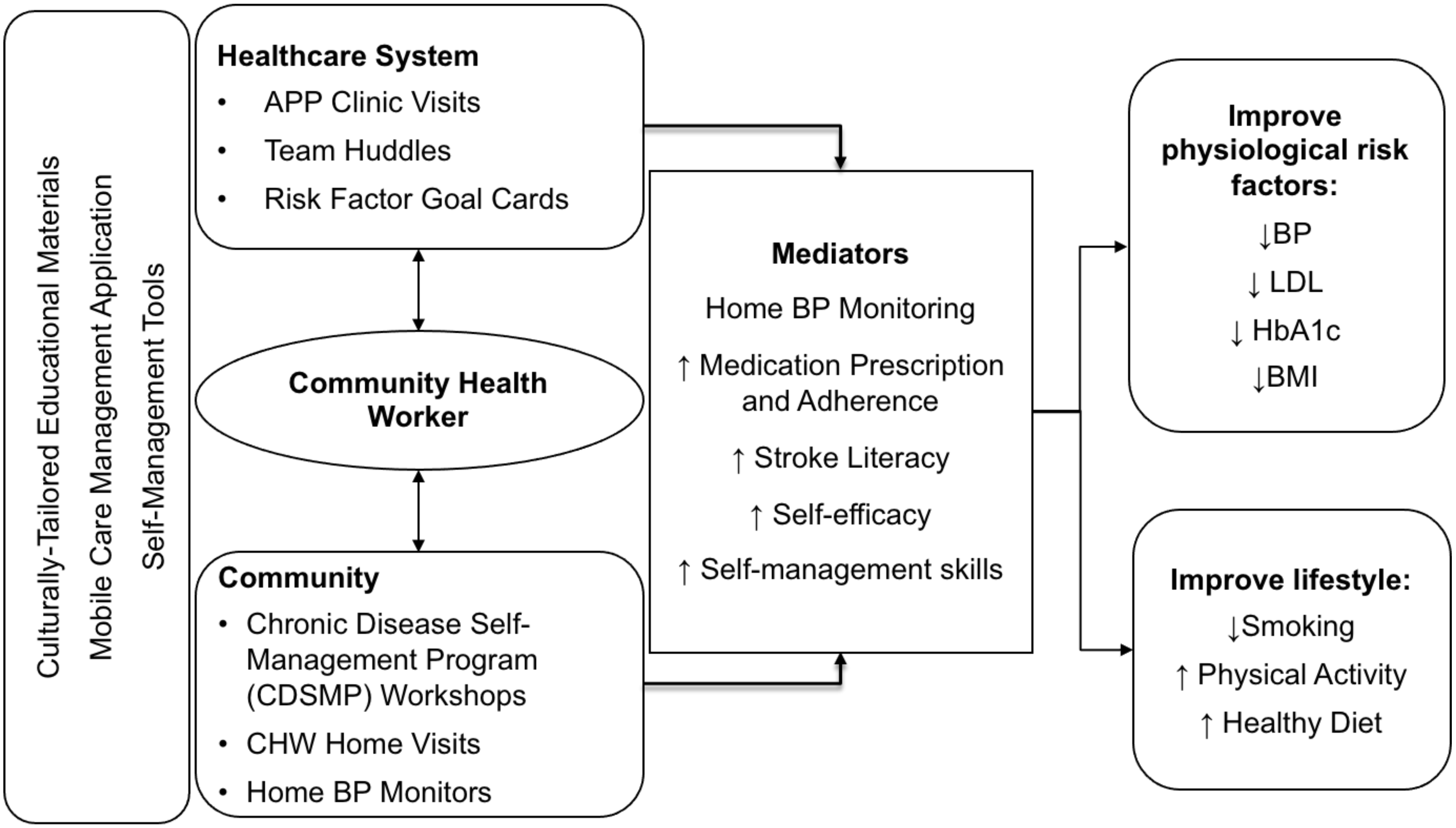

CHW: community health worker; APP: advanced practice provider; BP: blood pressure

© 2021 Towfighi A et al. *JAMA Network Open.*
