# Supplementary material for: Effect of a Coordinated Community and Chronic Care Model Team Intervention vs Usual Care on Systolic Blood Pressure in Patients With Stroke or Transient Ischemic Attack: The SUCCEED Randomized Clinical Trial
Source: JAMA Netw Open. 2021 Feb 15;4(2):e2036227. doi: 10.1001/jamanetworkopen.2020.36227 (PMC7885035; doi:10.1001/jamanetworkopen.2020.36227)
Supplement: Supplement 2. — Trial Protocol [file jamanetwopen-e2036227-s002.pdf]

FULL PROTOCOL

TITLE: Secondary stroke prevention by Uniting Community and Chronic care model teamEarly to End Disparities: the SUCCEED Trial

A multicenter, randomized controlled 500 subject trial of a Care Management and Community Health Worker led intervention to improve the management of stroke risk factors

MULTIPLE PRINCIPAL INVESTIGATORS

Barbara G. Vickrey, MD, MPH, Professor of Neurology & Vice Chair, Department of Neurology, UCLA

Amytis Towfighi, MD, Assistant Professor of Clinical Neurology
Department of Neurology, University of Southern California Chair of Neurology, Rancho Los Amigos National Rehabilitation Center

Supported by:

The National Institute of Neurological Disorders and Stroke (NINDS)

RFA-NS-12-007: Stroke Prevention/Intervention Research Program (SPIRP) (U54*)*

(Any modification to the SUCCEED protocol will be annotated on the coversheet or in an appendix. The annotation will note the exact words that are changed, the location in the protocol, the date the modification was approved by the Executive Committee, and the date it became effective.)

Version 1
April 2, 2012

TABLE OF CONTENTS

Page

SYNOPSIS .... 4 1.
STUDY OBJECTIVES 6

1.1 Primary Objective 6
1.2 Secondary Objectives 6

2. BACKGROUND 7

2.1 Rationale 7
2.2 Supporting Data 8

3. STUDY DESIGN 9
4. SELECTION AND ENROLLMENT OF SUBJECTS 10

4.1 Inclusion Criteria 10
4.2 Exclusion Criteria 10
4.3 Study Enrollment Procedures 11

5. STUDY INTERVENTIONS 14

5.1 Interventions, Administration, and Duration 14
5.2 Handling of Study Interventions 14
5.3 Concomitant Interventions 14
5.4 Adherence Assessment 14

6. CLINICAL AND LABORATORY EVALUATIONS 15

6.1 Schedule of Evaluations 16
6.2 Timing of Evaluations 17
6.3 Special Instructions and Definitions of Evaluations 17

7. MANAGEMENT OF ADVERSE EXPERIENCES 20
8. CRITERIA FOR INTERVENTION DISCONTINUATION 21
9. STATISTICAL CONSIDERATIONS 22

9.1 General Design Issues 22
9.2 Outcomes 22
9.3 Sample Size and Accrual 22
9.4 Data Monitoring 23
9.5 Data Analyses 23

10. DATA COLLECTION, SITE MONITORING, AND ADVERSE EXPERIENCE REPORTING 24

10.1 Records to be Kept.. 24

10.2 Role of Data Management... 24
10.3 Quality Assurance 25
10.4 Adverse Experience Reporting 25

11. HUMAN SUBJECTS 26

11.1 Institutional Review Board (IRB) Review and Informed Consent 26
11.2 Subject Confidentiality 26
11.3 Study Modification/Discontinuation 26

12. PUBLICATION OF RESEARCH FINDINGS .... 27 13. REFERENCES 28

70 **APPENDICES I. MODEL INFORMED CONSENT**

SYNOPSIS

Study Title

Secondary stroke prevention by Uniting Community and Chronic care model teams Early to End Disparities: the SUCCEED Trial

Objectives

The primary objectives of this study are as follows:

(1) To develop a *community-centered component* of a Chronic Care Model-based secondary stroke prevention intervention.

(2) To test the impact of a sustainable Chronic Care Model-based intervention on control of systolic blood pressure (primary outcome), composite stroke risk, and each individual stroke risk factor (secondary outcomes), among 500 adults (over 90% minority) recruited "early" - during or shortly after hospitalization for an ischemic or hemorrhagic stroke or TIA - at one of the four Los Angeles County safety net medical centers.

(3) To conduct a cost analysis of SUCCEED from the perspective of the Los Angeles County Department of Health Services, using intervention costs, cost equivalents of associated utilization of county system resources, and cost equivalents of observed and projected vascular events.

Design and Outcomes

The study design is a multi-center, randomized controlled trial. Patients will be randomized to either a Care Management and Community Health Worker led intervention versus Usual Care to improve management of stroke risk factors. The outcomes of this study will be assessed by a blinded Research Assistant. The total planned enrollment of this trail is 500 subjects. Subjects will be enrolled in the RCT for a period of 1 Year.

Figure 1. SUCCEED Study Design

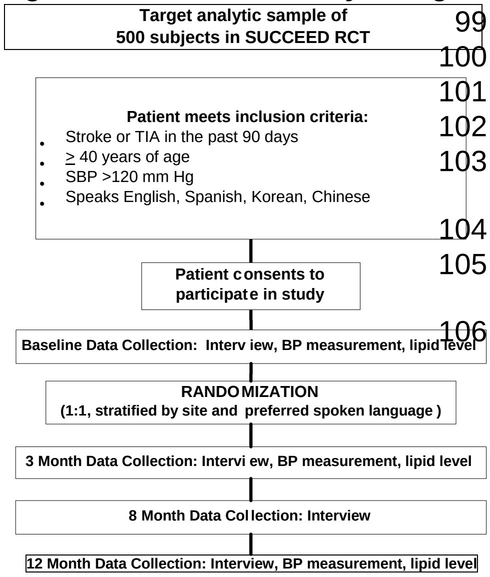

Interventions and Duration

We designate systolic blood pressure as the primary outcome for the purpose of sample size calculations. We will assess multiple relevant outcomes including all risk factors and a composite index, cost, quality of life, adverse consequences, moderators, mediators, and a range of metrics of intervention program implementation and participation.

Table 1 below shows the primary and secondary outcomes for SUCCEED.

Table 1. Primary & Secondary Outcomes

Primary Outcome
Blood Pressure
**Secondary Outcomes**
Risk of recurrent stroke, future cardiovascular event
Lipid profile: LDL cholesterol level, Triglyceride level, HDL cholesterol level
Hemoglobin A1c
C-reactive protein (CRP)
Adiposity: Body mass index (BMI), waist circumference, Waist-hip ratio, Waist-to-Height Ratio
Healthy Lifestyle Behaviors & Factors: Physical Activity, Diet, Smoking, Alcohol Use
Death

We will test the impact of this multi-level, multi-component intervention relative to usual care within the Los Angeles County public safety net healthcare system to improve management of stroke risk factors and increase adoption of healthy lifestyle habits. We will use culturally-adapted educational materials developed by 04/02/12 community-academic partnering teams in our Public Outreach and Dissemination Core. The on study period is 12 months. Additional follow-up off intervention will be every 6 months, up to 18 additional months during the period of center funding.

Data sources include:

- Examinations (Blood pressure, height and weight, waist and hip circumference, NIHSS for stroke severity, Rankin for disability): baseline, 3 months, and 12 months
- Fingerstick laboratory tests (lipid profile, Hgb A1c, CRP) baseline, 3 months, and 12 months
- Full survey: baseline, 3 months, 12 months
- Telephone survey: 8 months
- Telephone surveillance for vascular events and mortality: every 6 months, starting at 12 months, continuing for duration of center funding

Queries of the Los Angeles county administrative database will be conducted when the study is completed for LAC service and medication utilization.

Sample Size and Population

The setting of this study is all four county hospitals that anchor care for patients in the Los Angeles County public healthcare system (LAC), which serves the largest, most ethnically diverse county in the United States. This system, often called the Safety Net, serves more than 10 million residents and provides healthcare to 700,000 people every year, and treats more than 300,000 emergency and trauma victims every year. Through an integrated network of hospitals, health centers and clinics, the LA County Health Services System makes medical and preventive care services accessible in communities across the county. According to LAC administrative databases, more than 50% of patients use a language other than English as their primary language and 63% of outpatients are uninsured.

A total of 500 subjects with the onset of ischemic stroke, hemorrhagic stroke, or transient ischemic attack (TIA) within the prior three months will be recruited from the following off-campus locations:

- (1) Los Angeles County Hospital + USC Medical Center (LAC+USC),
- (2) Rancho Los Amigos National Rehabilitation Center (Rancho Los Amigos),
- (3) Olive View-UCLA, and
- (4) Harbor-UCLA Medical Center. No subjects will be recruited at UCLA.

Stratification variables are as follows, with 24 strata:

- Site (4 sites)
- Language (3: English, Spanish, other)
- Type of Stroke (2: ischemic/TIA, hemorrhagic)

1 STUDY OBJECTIVES

1.1. Primary Objectives

Primary Objective #1: To develop a *community-centered component* of a Chronic Care Model-based secondary stroke prevention intervention. This new component includes community health workers (CHWs) collaborating with healthcare system teams based at one of four Los Angeles County-Department of Health Services public “safety net” medical centers. CHWs - identified from the communities in which they serve - will receive specialized training and will conduct home visits to promote behavior change to address diet and physical activity risk factors, as well as assess for depression and social isolation. CHWs will use an existing, scalable mobile health platform specifically designed for care coordination and care teams to use by phone or iPAD, to assist patients in tracking selected clinical measures (blood pressure, lipids, weight/BMI, physical activity), to promote behavior change. CHWs will communicate and liaison with the healthcare provider team. The community-based component will *also* include cultural- and stroke-specific adaptation of physical activity and self-management programs at local community venues including health education and senior centers. *Hypothesis: We will develop a culturally-tailored intervention to improve stroke risk factor reduction.*

Primary Objective #2: To test the impact of a sustainable Chronic Care Model-based intervention on control of systolic blood pressure (primary outcome), composite stroke risk, and each individual stroke risk factor (secondary outcomes), among 500 adults (over 90% minority) recruited “early” - during or shortly after hospitalization for an ischemic or hemorrhagic stroke or TIA - at one of the four Los Angeles County safety net medical centers. We will conduct a randomized trial of this intervention that includes the new, communitycentered component developed in aim #1, and a “re-engineered” healthcare delivery system with nurse practitioners or physician assistants as care managers, distribution of home blood pressure monitors, group clinics, and a care management software system tailored to secondary stroke prevention and interfaced with the mobile health platform used by the CHWs, relative to usual care. We will include non-English speaking individuals from Hispanic, Chinese, and Korean racial and ethnic groups, and a broad range of stroke severity. We will analyze potential moderators (age, gender, stroke type and severity, education, country of birth/primary language, diabetes comorbidity, care team composition) to understand robustness of intervention impacts across levels of individual and health system characteristics. We will conduct a formative evaluation to continuously improve implementation, and will measure and analyze extent of intervention implementation, as it is delivered for up to 12 months after stroke or TIA through transition of patients into primary care. We will analyze potential mediators (life chaos, competing needs, medication adherence, knowledge of stroke risk factors, participation in self-management programs, social isolation, depression, health literacy, engagement with healthcare provider team, attitudes/health beliefs, self-efficacy, readiness to change, perceived risk, and perceptions of quality of interactions with provider) of the intervention’s impact, using both qualitative and quantitative methods.
Hypothesis: Patients randomized to the SUCCEED intervention will achieve 8mm greater reduction in systolic blood pressure reduction and lower stroke risk scores at 1 year versus those randomized to usual care.

Primary Objective #3:
To conduct a cost analysis of SUCCEED from the perspective of the Los Angeles County Department of Health Services, using intervention costs, cost equivalents of associated utilization of county system resources, and cost equivalents of observed and projected vascular events. *We will also develop a financial plan and proposal for sustainability* of the intervention if it is effective, incorporating data on cost and utilization, to describe potential financial arrangements or approaches – including reimbursement strategies - to support the intervention model in the future and in other settings. We will also produce training materials and protocols for future dissemination.

<u>Hypothesis</u>: If effective, the SUCCEED intervention will be sustainable because its community component was developed in partnership with the community and is culturally-appropriate, it will demonstrate a projected costoffset from future vascular events averted, and intervention tools and training programs will be codified.

1.2 <u>Secondary Objectives</u>

Not applicable

Version 1
04/02/12

2 BACKGROUND

2.1 <u>Rationale</u>

Stroke is the fourth leading cause of death and the leading cause of adult disability in the United States, with well-documented and substantial disparities in the prevention and outcomes of stroke for blacks, Hispanics, and Asians relative to whites. The strongest predictor of a future stroke is a prior stroke or transient ischemic attack (TIA). Although the risk of stroke can be substantially reduced through control of modifiable risk factors including hypertension, diet, physical activity, and smoking, and through optimal management of diabetes and dyslipidemia there is abundant evidence that these risk factors are under optimal control in only a small proportion of stroke survivors, exposing hundreds of thousands of individuals each year to preventable death and disability from subsequent strokes. The situation is even more pressing among indigent, minority populations, in which knowledge about stroke is limited and barriers to accessing healthcare – transportation, healthcare-related costs, screening of phone communication due to concerns about immigration authorities, bill collectors, inability of healthcare providers to maintain current phone numbers due to patients use of prepaid phones that run out, beliefs about causes of stroke symptoms, unhealthy diets, lack of safe places to walk or local stores with fresh produce, homelessness, and many others - are formidable. Our group previously documented gaps in prevention care after stroke and analyzed barriers among the large, multi-ethnic indigent population in Los Angeles County, where out of the entire county's approximately 10 million residents spread over several thousand square miles, fewer than 30% are non-Hispanic whites.

Based on International Classification Codes (ICD) codes generated from the Los Angeles county Department of Health Services (LAC-DHC) database, there are over 1000 admissions of stroke per year at the four County anchor hospitals. This eligible population is more than 20% greater than in SUSTAIN due to the inclusion of hemorrhagic stroke. The eligibility is further increased by including more languages spoken by subjects.

Table 2. Stroke and TIA patients hospitalized in Los Angeles County, 2009-2011

Facility	Year	Stroke ICD: 433,434, 436	TIA ICD:435	Hemorrhagic Stroke ICD: 430, 431	Total
Harbor-UCLA Medical Center	2009	250	65	72	387
	2010	218	58	72	348
	2011	250	57	59	366
Olive View Medical Center	2009	124	31	7	162
	2010	178	29	8	215
	2011	131	30	4	165
	2009	148	46	11	205
Rancho Los Amigos	2010	166	40	12	218

	2011	143	45	18	206
	2009	192	31	98	321
LAC+USC	2010	192	52	119	363
		184	51	116	
	2011	714	173	188	351
All Los Angeles County Safety Net Hospital	2009				1075
	2010	754	179	211	1144
	2011	708	183	197	1088

In addition to this eligible inpatient pool, outpatients who have had the onset of with a recent history of stroke or TIA are also eligible. Rancho Los Amigos is implementing a medical home model for uninsured patients with a recent stroke, so patients who were admitted elsewhere for their stroke can be fast-tracked to receive postdischarge follow-up care at Rancho Los Amigos.

Starting in 2010, Los Angeles County has implemented an Emergency Medical System (EMS) routing policy. Patients with acute onset of stroke symptoms within three hours are diverted to stroke certified centers. None of the four LAC hospitals have obtained this designation yet. However, we do not anticipate this to be a barrier to enrolling enough patients because the number of hospitalized stroke and TIA patients in 2011 remains above 1000.

2.2 **Supporting Data** Our research team has designed and is implementing a chronic care model-based program called SUSTAIN (Systemic Use of STroke Averting INterventions) to improve the delivery of secondary stroke preventive services after hospital discharge. This care intervention includes group clinics, self-management support, report cards, decision support through care guides and protocols, and coordination of ongoing care. The first specific aim is to test via a randomized-controlled trial whether SUSTAIN improves blood pressure control among an analytic sample of 268 patients with a recent stroke or transient ischemic attack discharged from four Los Angeles County public hospitals. Secondary outcomes consist of control of other stroke risk factors, lifestyle habits, medication adherence, patient perceptions of care quality, functional status, and quality of life. A second specific aim is to conduct a cost analysis of SUSTAIN from the perspective of the Los Angeles County Department of Health Services, using direct costs of the intervention, cost equivalents of associated utilization of county system resources, and cost equivalents of the observed and predicted averted vascular events.

In this study, we build upon our experiences implementing SUSTAIN to enhance the access to community resources. These include the use of community health workers, mobile technology to synchronize information while in the field, and increasing access to self-management classes.

3 STUDY DESIGN

Figure 1. SUCCEED Study Design

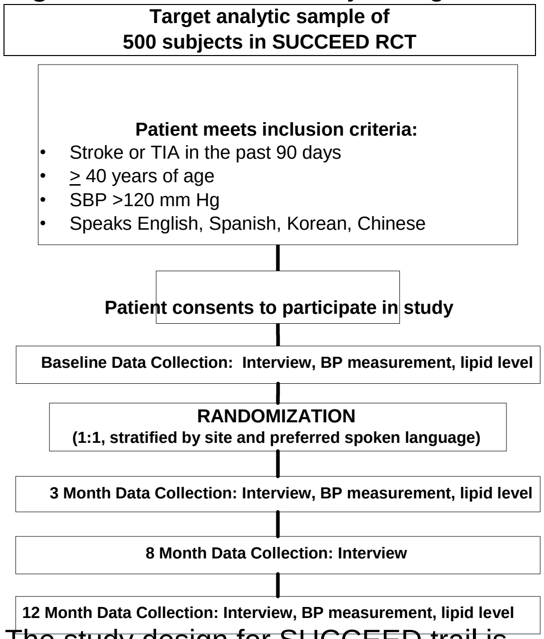

The study design for SUCCEED trail is a multi-center, randomized controlled trial of a Care Management and Community Health Worker led intervention VS Usual Care to improve management of stroke risk factors. The outcomes of this study will be assessed by a blinded Research Assistant. Subjects will be enrolled in the RCT for a period of 1 Year. A diagram of the study is provided as shown in **Figure 1 to the left**.

We designate systolic blood pressure as the primary outcome for the purpose of sample size calculations. We will assess multiple relevant outcomes including all risk factors and a composite index, cost, quality of life, adverse consequences, moderators, mediators, and a range of metrics of intervention program implementation and participation.

4 SELECTION AND ENROLLMENT OF SUBJECTS

The inclusion and exclusion criteria for the SUCCEED RCT are shown below:

Table 3. Inclusion and Exclusion Criteria of RCT

Inclusion Criteria
• Patients of LAC + USC, Rancho Los Amigos, Olive View-UCLA, or Harbor-UCLA with onset of ischemic stroke, hemorrhagic stroke, or TIA within the prior three months.
Exclusion Criteria
• Age < 40 years<br>• SBP < 120 mm Hg<br>• Speaks language other than English, Spanish, Korean, Mandarin, and Cantonese<br>• Cannot provide informed consent due to dementia or aphasia

4.1 Inclusion Criteria

4.1.1 *The disease or disorder under study, and how it is being documented (diagnostic methods, criteria for evaluation)*
Patients with onset of ischemic stroke, hemorrhagic stroke, TIA within the prior three months will be included in SUCCEED. Diagnosis is confirmed by referring clinician.

4.1.2 *Clinical indicators of currents status, as measured with XX days of randomization*. Not applicable

4.1.3 *Prior therapy, if any. Consider listing prior treatments. Consider listing the allowable duration for the specific population to be studied (treatment-naïve, treatment-experienced, or prior-treatment-failed "salvage subjects")* Not applicable

4.1.4 *Demographic characteristics*
Patients over 40 years of age will be included in SUCCEED. Patients will be included if they speak English, Spanish, Korean, Mandarin, or Cantonese.

4.2 Exclusion Criteria

4.2.1 *List any clinical contradictions. Specify grades of signs/symptoms*
Persons with language (aphasia) or cognitive difficulties are ineligible if they cannot communicate that they understand the study during the informed consent process.

4.2.2 *Clinical/laboratory indicators of current status, obtained within XX prior to randomization. List the specific tests to be performed and the narrowest acceptable range of laboratory values for exclusion, consistent for safety.*
Systolic Blood pressure > 120 mmHg

4.2.3 *Specify any exclusion related to pregnancy, lactation, or plans to become pregnant. Specify methods for assessing current status and willingness to use contraception, if applicable.* Not applicable

4.2.4 *Use of excluded drugs, devices, etc. within XX days prior to entry* Not applicable

4.2.5 *For drug studies: Allergy/sensitivity to study drugs or their formulations* Not applicable

4.2.6 *Specify any clinical (life expectancy, co-existing disease), demographic (age) or other characteristics that precludes appropriate diagnosis, treatment, or follow-up in the trail*

Young patients are excluded because the mechanism for their strokes is frequently not due to atherosclerosis (e.g. arterial dissections, congenital coagulation defects, etc), so the interventions in this proposal may not be as applicable to them. We will also exclude persons who already meet the primary outcome of SBP <120 mm Hg. We will not be able to translate written materials in languages other than English, Spanish, Korean, and Chinese. Patients who do not speak any of those languages will be excluded.

4.2.7 *Active drug or alcohol use or dependence that, in the opinion of the site investigator, would interfere with adherence to the study requirements.*

If the participant exhibited active drug or alcohol use or dependence that, in the opinion of the site investigator would interfere with adherence to study requirements, that participant would be excluded from the study.

4.2.8 *Serious illness (requiring systemic treatment and/or hospitalization) until subject either completes therapy or is clinically stable on therapy, in the opinion of the site investigator, for at least XX prior to the study. (List specific illnesses and acceptable time)*

Not applicable, hospitalized patients are being enrolled in the study.

4.2.9 *Inability or unwillingness of subject to give written consent.*

If a subject is unwilling to give written consent, they will be excluded from the trial. Persons with language (aphasia) or cognitive difficulties are ineligible if they cannot communicate that they understand the study during the informed consent process.

4.3 <u>Study Enrollment Procedures</u>

4.3.1 *Methods for identifying and recruiting candidates for the trial*

The research team will present SUCCEED to clinicians in the neurology, internal medicine, family medicine, emergency department, and the rehabilitation departments. Study flyers will be posted on the wards, outpatient clinics and housestaff offices of these departments. In the inpatient setting, we will discuss the study with neurology ward nurses and discharge planners, and they will include study flyers in discharge packet. In the outpatient setting, we will discuss the study with the clinic coordinators. Housestaff will be given pocket-sized index cards about the trial. One side of the index card contains a short description of the aim and the eligibility requirements. The back side of the index card contains an IRB-approved short script of a few sentences that describe the study and ask for verbal permission for a member of the research team to approach them to further discuss the study. Finally, contact information of the research team is listed on the card.

We will adapt our highly successful protocol utilized in the SUSTAIN study to identify, approach, and recruit subjects for the SUCCEED study. As stated earlier, a potential subject's clinician need to obtain verbal permission from them before passing their name to a member of the research team.

In the inpatient setting, we have found that embedding the RA within the inpatient team during rounds fosters awareness about the study and prompts the study team during rounds to verbally ask patients for permission for the RA to meet the patient later.

In the outpatient setting, clinicians would notify the site PI, who attends the clinic. The Site PI then contacts the research team RA. Because outpatients may need to leave the clinic immediately after the visit with the clinician, the RA may not be able to speak to outpatients before they leave, but they will be enabled to call the patient at a later time.

Alternatively, potential subjects can also directly call the RA using the toll-free number listed on flyers to learn more about the RCT.

4.3.2 *Procedures (e.g., maintaining a screening log at each clinical site) for documenting how subjects learned about the trial, who referred them to the trial, reasons for ineligibility, and reasons for nonparticipation of eligible subjects.*

Please refer to section 4.3.3 for procedures on consent, documenting how subjects learned about the trial, who referred them to the trial, reasons for ineligibility, documentation of ineligibility, and reasons for nonparticipation of eligible subjects. A description of how this information will be collected centrally and used to enhance subject recruitment efforts is also provided.

4.3.3 *Consent (and assent) procedures*

<u>Interaction between the RA and the potential research subject</u>

- *Arranging initial meeting*: When a clinician notifies the RA a face-to-face meeting will be arranged to check whether the patient meets eligibility criteria for enrollment and to describe elements required for informed consent. The RA will read an IRB-approved script that further describes the SUCCEED trial to the patient.

  If a potential subject directly contacts the RA using the toll-free number, the RA will read the IRBapproved script that further describes the SUCCEED trial to the patient. If the potential subject is interested, a face-to-face meeting will be scheduled.

- *Setting:* The consent process will take place in a private room. If the meeting takes place in a shared hospital room, the research staff member will close the curtain when meeting with the participant. The RA will describe the study. RA will be bilingual in English or Spanish, but in the case that a subject speaks a language other than English or Spanish, the research team will use volunteers fluent in the appropriate language to translate for the RA.

- Eligibility: If interested in continuing, the RA will ask potential subjects questions about age, and onset of stroke to confirm eligibility. They will also take BP measurements. Finally, they will ask questions that require comprehending the basics details of the study. If participants cannot describe what is told to them, they will not be enrolled in SUCCEED. For potential subjects found to be ineligible, the reasons for ineligibility will be recorded.

- Voluntary consent: The RA will ensure that eligible subjects are given every opportunity ask questions, and remind the patients that participation in SUCCEED is voluntary. If interested, the potential subject will sign the written consent form. Potential participants will have the opportunity to take the consent document home and discuss participation with others before making a decision.

- Declining consent: If patients do not consent to the study, the RA will record the reason why the patient did not consent. Further, the RA will ask permission to use the demographic information of the patient to generate enrollment propensity weights. Enrollment propensity weights will be used to analyze how the tendency to participate in the RCT impacts attainment of outcomes.

- Storing consent forms. The consent forms at each site will be stored in a locked file cabinet within a locked room. Access is limited to the site’s Principal Investigator and research staff.

For the baseline survey, the RA will enter the circumstances of the initial visit (inpatient/outpatient, whether clinical team called RA or subject, reasons for ineligibility, and reasons for nonparticipation). These data will be summarized in a monthly report by site and shared among the Task Force and the research teams at UCLA and at each site.

4.3.4 *Procedure for obtaining intervention group assignment*

Before the RCT begins, we will use computer-assisted stratified randomization of block size of 4. The three stratification variables will be site, spoken language (Spanish, English or other), and type of stroke (ischemic or hemorrhagic). These lists have an allocation ratio of 1:1 of control and intervention. Permuted block randomization that stratifies by site will be used to promote periodic balance through the trial and group balance at the end of the trial. The study programmer/analyst will generate 24 lists, one for each stratum. Each row of the list will include a unique subject IDs and intervention arm of the study. The lists will be kept in a notebook that the Project Coordinator will possess at all times.

For patients who consent to the study and meet inclusion criteria, the RA will collect baseline data (described below in section of Outcome Measures) before randomization so that the RA will collect data uniformly regardless of the eventual allocation of the subject. After an eligible participant has consented and completed the baseline survey, the RA will inform the Project Coordinator with the information needed to choose which of the 24 lists to use. The Project Coordinator will find the next uncrossed row, tell the RA the unique subject ID and intervention assignment, then cross out that row so that the next row will be used when this list is used again.

The Project Coordinator will assign the newly enrolled participant a unique subject ID and inform the RA the assigned randomization status of this subject. The Project Coordinator will mark down in the notebook the date of randomization for that subject ID.

<u>Efficiency and productivity metrics such as tracking the effectiveness and outcomes of recruitment strategies employed</u>

We have the data to determine the enrollment ratios.

- Numerator: we have developed tracking databases for the RAs to record the number of approached patients per month. They further document the reason for ineligibility among subjects. For eligible subjects, the RAs further document which patients consent or decline to participate, and for those that decline, the reasons for not participating.
- Denominator: On a periodic basis, administrators at the LAC-DHS run reports for the SUSTAIN team that show the number of patients hospitalized at each site per month.

Using our tracking databases and the LAC-DHS reports, we can calculate the proportion of hospitalized stroke patients approached by our research team and the proportion of eligible stroke patients who consent to participate in the study. This will allow us to discern when and where and why study enrollment rates are lower than expected.

These enrollment statistics will be shared among the research team and the Task Force. If rates of participating are lower than expected at a site, we will investigate the reasons for doing so and develop strategies for overcoming barriers. This feedback loop has been critical for developing our successfully recruitment strategy in SUSTAIN.

5 STUDY INTERVENTIONS

5.1 <u>Interventions, Administration, and Duration</u> The intervention team consists of the following:

- Care Managers
- Community Health Workers
- Research Assistants

The intervention is:

- Care by CM
- Medical algorithms
- Report cards

Community Health worker home visits and community self management classes, supported by mobile technology.

5.2 Handling of Study Interventions

Not applicable **5.3 Concomitant Interventions**

5.3.1 *Required Interventions* Not applicable

5.3.2 *Prohibited Interventions* Not applicable

5.3.3 Precautionary Interventions Not applicable

5.4 Adherence Assessment

For subjects randomized to the intervention arm, we will document the number of encounters between subjects and the intervention team. This will include the number of face-to-face visits, number of telephone calls, number of classes attended, and the number of home visits.

6 CLINICAL AND LABORATORY EVALUATIONS

The Schedule of Evaluations in section 6.1 indicates all study evaluations. An ‘X’ in a cell indicates that a particular evaluation is to be performed at a particular study visit.

The definitions for the Schedule of Evaluations are included in section 6.2 where evaluations are defined, timelines are provide, special considerations or instructions for evaluations are included.

477

6.1 Schedule of Evaluations

		On-Intervention			Off-Intervention		
**Evaluation**	**Screening/ Baseline**	**3 months**	**8 months**	**12 months**	**18 months**	**24 months**	**30 months**
**Informed Consent**	X						
**Physical Exam**: Blood Pressure (BP), Body Mass Index (BMI), Waist-Hip Ratio, Waist-toHeight Ratio, NIH Stroke Severity Scale, Modified Rankin Scale	X	X	X	X			
**Finger Stick LDL** (Triglyceride level, HDL cholesterol level, Hemoglobin A1c, C-reactive protein (CRP)	X	X	X	X			
**Full In-Person Questionnaire**	X	X		X			
**Brief Telephone Questionnaire**			X				
**Telephone Surveillance (vascular events/death)**					X	X	X

6.2 Timing of Evaluations

6.2.1 *Pre-Randomization Evaluations*

*Please note: All evaluations occur prior to the subject receiving any study interventions.

Screening, Entry Surveys, and Randomization

Screening for inclusion/exclusion criteria, baseline (entry surveys), and randomization occur in the same face-to-face encounter.

6.2.2 *On-Study/On-Intervention Evaluations*

See Section 6.1 for the schedule of evaluations occurring after randomization while the subject is onstudy. The allowable window for evaluations during the on-intervention period is one month around each timepoint.

6.2.3 *Intervention Discontinuation Evaluations*

If patients wish to withdrawal from the study prematurely, we will attempt to administer the final outcome measurement survey. Additionally, the reason for discontinuation will be recorded.

Retention of subjects will be enhanced by collecting multiple phone numbers and addresses for each subject and multiple phone numbers for a close friend and family member.

6.2.4 *On Study/Off-Intervention Evaluations*

The evaluation schedule remains the same while the subject is “off intervention/on study” for SUCCEED (see Section 6.1).

6.2.5 *Final On-Study Evaluations*

The schedule of activities during the final 12 month measurement time point is the same as the baseline and 3 month time points, with the difference being that there is no need to conduct the randomization procedures other than the baseline time points.
After the study is completed, we will call the subjects every 6 months for up to 30 months after randomization to conduct surveillance of vascular events and ascertainment of mortality.

6.2.6 *Off-Study Requirements*

Once the subject has completed the protocol-specified period on study intervention, they will be followup with every 6 months for up to 18 months for the occurrence of vascular events and death. Refer to Section 6.1.

6.2.7 *Pregnancy*

Women who become pregnant while on-study, will be instructed to continue their normal care. No additional evaluations are needed.

6.3 Special Instructions and Definitions of Evaluations

6.3.1 *Informed Consent*

The patient education and informed consent process has been detailed in Section 4.3.3. Further, plans review of consent document in case changes may be required and how documentation of signed consent will be maintained by the study are outlined in that section.

A model informed consent form should has been included as Appendix. When developing the consent form, consider including language allowing for the retention of study data and specimens beyond the close of this study, for sharing the de-identified data and specimens with other researchers, and for using the specimens for purposes beyond the scope of this study.

6.3.2 *Documentation of [specify the Disease/Disorder under study]*

We will review administrative databases to determine if the patient has suffered a stroke, myocardial infarction, and death during the study period.

6.3.3 *Medical History*

Self report in a survey. We will review administrative databases to determine if the patient has suffered a stroke, myocardial infarction, and death during the study period.

6.3.4 *Treatment History*

Self report in a survey

6.3.5 *Concomitant Treatments*

Not applicable

6.3.6 *Study Intervention Modifications*

Not applicable

6.3.7 *Clinical Assessments*

Because NINDS strongly encourages investigators to make use of the NINDS Common Data Elements in developing the CRFs (see http://www.commondataelements.ninds.nih.gov/), the study team will rely on those materials in the development of CRFs for SUCCEED.

Blood Pressure (BP): Participants are seated quietly for 5 minutes before Blood Pressure (BP) reading are initiated. Staff are trained to ensure that the proper cutoff size is used and that the participant's arm is resting on a flat surface, with the BP cuff at heart level. Two sequential readings will be taken using the OMRON 90XL with 5 minute intervals between each reading. If the first two readings differ by more than 5mmHg, then an additional two readings are taken and recorded electronically using a laptop based data entry system developed through Core A. Average BP is calculated by the data collection system.

NIHSS and disability scales: Each RA will obtain certification to perform the National Institute of Health Stroke Scale (NIHSS) and the modified Rankin scale prior to the start of the study. These are measures of stroke severity and disability. Certification is obtained by completing the following courses and taking a test by scoring an online video of a patient. http://nihss-english.trainingcampus.net/uas/modules/trees/windex.aspx and http://rankin-english.trainingcampus.net/uas/modules/trees/windex.aspx?res.
In addition, the RA will be observed by the site PI for at least the first two study participants. Each RA will be re-observed examining a study participant 2 months later, then 4 months later.

6.3.8 *Laboratory Evaluations*

LDL Cholesterol: Point-of-service CardioChek meters will be used to collect information on LDL cholesterol. The meters require only a small capillary blood sample (from a fingerprick). After cleaning

the end of one finger with an alcohol wipe, a small lancet is used to prick the participant's finger. The first blood spot is wiped off with gauze and the next blood spot is dropped into a special cartridge which is then simply inserted into the CardioCHek meter for the calculation of LDL cholesterol. Staff will record results electronically using the laptop-based data entry system.

Glycosylated Hemoglobic (HgA1c) and C-Reactive Protein (CRP): We will use dried blood spots (DBS) to obtain assays for these two parameters. Whatman 903 Protein Saver filter paper is used for DBS collection; this paper is commercially available as small cards. Participants should be well hydrated as this facilitates collection of adequate DBS. Staff are trained to ask about hydration and to provide some water if participant hasn't had much to drink recently. Participants are asked to wash their hands with very warm water, to remove lotions, and to aid blood flow. To further aid in blood flow to the hand, participants can be asked to wrap their hand in a heating pad or two hold a heat pack and/or to hang their arm down and shake their hand downward/ Similar to the CardioChek protocol, the end of one finger is then cleaned with an alcohol wipe, a small lancet is used to prick the middle or ring finger on the fleshy side. The first blood spot is wiped off with gauze. The following blood spot is allowed to "pool" on the finger and then to drop onto the collection card. (Whatman cards have circles printed on the card so blood spots can be dropped to fill-in each circle) Cards will be labeled with each subject's study ID, along with information on date and time of collection. Once blood spots have been collected, cards are placed as "drying boxes" as they require air for at least 2 hours. With cards individually "housed" in separate boxes, staff can stack boxes of cards over the course of a day as they collect samples from participants. Once cards have dried at least 2 hours, they are to be placed in a storage envelope with a dessicant packet (all supplies provided through Core C) and then frozen. Samples collected for Project 1 at hospital settings will be stored in local freezers and transported weekly to Core C freezers on the UCLA campus.

6.3.9 *Pharmacokinetic Studies*

Not applicable

6.3.10 *Other Laboratory Studies*

Not applicable

6.3.11 *Additional Evaluations*

Not applicable

6.3.12 *Questionnaires*

Full in person Questionnaires:

Demographics, Stroke knowledge/attitudes, Medication adherence (simony scale), Competing needs, Access to care, Chaos questions (Outreach questionnaire), Perception of Neurological care (CHAHPS), Patient Health Questionnaire (PHQ2), Social support items, Medical Utilization items (including hospital and physicians visits and other medical services), RAND SF-6D

Brief Telephone Questionnaire:

Medication adherence (simony scale), Medical Utilization items (including hospital and physicians visits and other medical services)

6.3.13 *Adherence Assessments*

For subjects randomized to the intervention arm, we will document the number of encounters between subjects and the intervention team. This will include the number of face-to-face visits, number of telephone calls, number of classes attended, and the number of home visits.

7 MANAGEMENT OF ADVERSE EXPERIENCES

- Elder Abuse: Under California law, all healthcare employees are mandated to report elder abuse. If abuse is suspected during your encounter with the patient, the site PI will be contacted.

- Elevated Blood Pressure: If the subject experiences elevated blood pressure that meets the following criteria, the following steps should be taken:
    - If ≥ 140 – verbally inform the subject that their BP is at least 140 and give a card to the subject with the reading.
    - If ≥ 180 – inform the subject that they must see their doctor immediately. If the subject does not have a primary doctor, the patient must go to the emergency room.

- Suicide: Although the subject will not be asked specific questions regarding suicidality, if the subject expresses suicidal ideation or states they are thinking about harming themselves or have recently attempted to do so, the site PI will be contacted.

8 CRITERIA FOR INTERVENTION DISCONTINUATION

If a patient has optimal control of all risk factors, the criteria for discontinuing intervention are met, but that participant will still remain a part of the study.

9 STATISTICAL CONSIDERATIONS

9.1 General Design Issues

The primary and secondary hypotheses is that subjects randomized to the intervention arm will have better control of risk factors and higher rates of adoption of healthy lifestyle habits compared to the control arm.

All outcomes will be collected by a blinded RA. Vascular events will be collected by patient self-report and will not require an adjudication committee.

The design is a RCT, 1:1 allocation to control and intervention arms. There is no crossover.

Randomization is stratified by site (4), language (3: English, Spanish, other), and type of stroke (2: ischemic/TIA, hemorrhagic). There are 24 strata.

Intervention on period is up to 12 months for persons. It may be discontinued if subjects optimally control risk factors.

After the 12 month study period, subjects will be monitored by telephone for the presence of vascular events and mortality. There is no face-to-face contact, and thus, no collection of SBP, the primary endpoint. This surveillance is to better determine the impact of the intervention in the first 12 months after randomization.

9.2 Outcomes

9.2.1 *Primary outcome*

Refer to **Table 1** provided below.

9.2.2 *Secondary outcomes*

Refer to **Table 1** provided below.

Table 1. Primary & Secondary Outcomes

**Primary Outcome**
Blood Pressure (change in blood pressure, as well as proportion of patients with BP under 120/80)
**Secondary Outcomes**
Risk of recurrent stroke, future cardiovascular event
Lipid profile: LDL cholesterol level, Triglyceride level, HDL cholesterol level
Hemoglobin A1c
C-reactive protein (CRP)
Adiposity: Body mass index (BMI), waist circumference, Waist-hip ratio, Waist-to-Height Ratio
Healthy Lifestyle Behaviors & Factors: Physical Activity, Diet, Smoking, Alcohol Use
Death

9.3 Sample Size and Accrual

Power Analysis

Power Analyses was conducted with comparison of main outcomes (systolic blood pressure (SBP)) between the control and intervention arms. Based on availability of patients and our capacity, we plan to enroll 500 subjects from four senior centers. Using Intra-class correlation (ICC) of the four centers at 0.0085 level, attrition after the baseline between 20% to 30% level, mean SBP 141 mm Hg and standard deviation (SD) 20, three

repeated measurements: baseline, 3 months, and 12 months (Brown and Prescott 2006), the underlying statistical power of the study comes from two dimensions of observations: the number of unique subjects and the number of repeated measurements within a subject. Based on sample size and power analyses method proposed by Diggle et al. (1994) and Liu et al. (2005) for repeated measures analysis with a type I error of 0.05, type II error of 0.2 (or equivalent to power of 80%), 2.6 and 2.4 average data points for each subject (corresponding to 20% and 30% attrition respectively), and an auto-correlation at 0.2 level, the effective sample size will be 147 and 147 out of the planned 250 and 250 participants in each of the 2 arms (after adjusting for clustering effect), which will enable us to detect an effect size for SBP as small as 0.215 in standard deviation units or 4.29 mm Hg (for 20% attrition), and 0.253 in standard deviation units or 5.06 mm Hg (for 30% attrition).

The seminal work for moderator and mediator effect was from Baron and Kenny (1986). For potential moderators (e.g., age, gender, stroke type and severity, education, country of birth/primary language), we will test power of moderation using the most common measure of effect size f^2 (Aiken & West, 2001), which equals the unique variance explained by the interaction term divided by sum of the error and interaction variances. Cohen (1988) has suggested that f^2 effect sizes of 0.02, 0.15, and 0.35 are termed small, medium, and large, respectively. A more realistic standard for effect sizes with moderators might be 0.005, 0.01, and 0.025 for small, medium, and large, respectively.

Due to the low power in tests of moderation, there are greater interests in mediation over moderation (McClelland & Judd, 1993). To analyze power of potential mediators (e.g., self-efficacy, self-management, social isolation, depression, perceived risk of stroke), we will use indirect effect, which is the product of coefficient from independent variable to mediator, and mediator to the outcome. Because an indirect effect is a product of two effects, the usual Cohen (1988) standards of .1 for small, .3 for medium, and .5 values should be squared. Thus, a small effect size would be .01, medium would .09, and large would be .25 (or we can use small .02, medium .15, and large .40.)

The effect size for SBP as small as 0.215 in standard deviation units or 4.29 mm Hg (for 20% attrition), and 0.253 in standard deviation units or 5.06 mm Hg (for 30% attrition) can be considered clinically meaningful in the present of both moderators and mediators. Thus, the planned sample size should be sufficient for analyses.

9.4 Data Monitoring

For Care Management studies such as this proposed study, a Data Safety Monitoring Board (DSMB) is not usually required. However, should NINDS require a DSMB, we will establish an Internal DSMB for this minimal risk study. Care coordinators will be monitoring risk factor control and resource utilization of subjects randomized to the intervention. Care coordinators will be making multiple telephone calls about coordination of care. They will ask subjects about the steps they are taking to lower the risk of stroke.

ClinicalTrials.gov Requirements

This trial does not meet the FDAAA requirement for mandated registration in ClinicalTrials.gov because it does not involve a drug, biologic, or device. However, we do plan to register this trial on ClinicalTrials.gov. Such registration is required for findings to be published in many medical journals, and it could also serve to enhance the dissemination of the trial's results.

9.5 Data Analyses

Baseline characteristics between the SUCCEED intervention and usual care groups will be compared.

Continuous variable means will be compared using the t-test and ordinal or non Gaussian continuous variables will be compared using the Wilcoxon rank sum test. Unordered categorical variables will be compared using Chi-square test or Fisher's exact test.
Enrollment weights based on a logistic regression model will be calculated using demographic data collected from eligible non-participants. If needed, attrition weights will be determined from logistic regression models using demographic data on participants who drop out of the study. These two weights will be combined to form an overall weight (using the inverse of the product of the probabilities of participation). Both raw and adjusted rates using the overall weight will be compared between SUCCEED and usual care arms.

Intention to treat analyses on all primary and secondary outcomes will be conducted using ordinal logistic or multiple linear regression models, incorporating the overall weight. Intervention status will be an independent variable in all models. We will compare study outcomes between the control and intervention groups both with and without adjusting for potential covariates associated with the outcome measure.

We will test the moderator and mediator effects separately. To assess the moderating effects, we will compare regression models we fit including interaction terms between the independent variables and each possible effect modifier, and the models that do not include the corresponding interaction terms, using the likelihood ratio test (LRT). To assess the mediating effects, we will use Baron and Kenny (1986) methods to run a series of regression analysis and use the Sobel test and Bootstrapping to test mediating effects.

To check the fidelity (uptake) of the SUCCEED intervention, we will also analyze attendance at SUCCEED clinics and the number of telephone coordination of care calls made during enrollment.
Because subjects will be recruited from four hospital systems, there could be some underlying intra-site correlations of outcome measures in the collected data, which will have potential impacts on statistical significant tests of parameter estimates in the analyses. To take this into account in our analyses and modeling, robust standard errors will be calculated using Huber/ White/Sandwich method to ensure valid significance testing. Although we will assess for the impact of clustering data structure, we do not expect a large intra-site correlation because the three sites are part of the same county healthcare system and we are not aware of differences of outcomes by site. Furthermore, we will also include site dummies in our model to control and evaluate the differences in parameter estimates, especially if there are significant variations among the sites.

10 DATA COLLECTION, SITE MONITORING, AND ADVERSE EXPERIENCE REPORTING

10.1 <u>Records to Be Kept</u>

<u>Linking file</u>
Each of the four sites will maintain a linking file that includes identifying information (name, address, phone number) and the subject ID. These files are for the purpose of subject tracking, and subject payment tracking. These files will be password-protected on a designated computer in a locked office and only site's Principal Investigator and research staff will have access to the tracking file. Site's tracking files will not be sent to UCLA. Only the site's Principal Investigator and research staff will have access to the tracking files. Electronic files will be kept until the completion of all data collection and the successful creation of the final analytic dataset.

10.2 <u>Role of Data Management</u>

10.2.1 *Clinical site responsibilities in data collection and management*

The clinical site responsibilities are to perform the examination, laboratory tests, and the surveys on the subject.

They will then enter the values directly into the web database of outcome measures. The research team will enter a username and password to generate a blank form for entering

outcome data. The RA will enter the subject ID and the measurements from the examination, survey, and laboratory procedures. Once the data is submitted, the data is not retrievable on the internet, but only to the research team at UCLA. After the data is entered, it is longer available to persons at the site. It will only be accessible at UCLA, the Statistical Center for the study.

10.2.2 *Statistical Center responsibilities in data management*

The Statistical Center will analyze the data stored on a UCLA server. No identifiers that will be kept with the data, so it is considered anonymous.

10.3 Quality Assurance

The central research team at UCLA will periodically conduct site visits to ensure quality assurance, and to check if evaluations are collected appropriately. Additionally, the data will be reviewed periodically by the central research team at UCLA to audit data for completeness.

10.4 Adverse Experience Reporting

Adverse experiences will be reported as required by the IRB at each site. There are no additional FDA regulations because we are not studying a biological agent. The Manual of Operations will include detailed definitions of adverse experiences, a table for grading their severity, and details of how clinical sites are to report them.

11 HUMAN SUBJECTS

11.1 Institutional Review Board (IRB) Review and Informed Consent

This protocol and the informed consent document (Appendix I) and any subsequent modifications will be reviewed and approved by the IRB or ethics committee responsible for oversight of the study. A signed consent form will be obtained from the subject. The consent form describes the purpose of the study, the procedures to be followed, and the risks and benefits of participation. A copy of the consent form will be given to the subject, and this fact will be documented in the subject's record.

11.2 Subject Confidentiality

All laboratory specimens, evaluation forms, reports, and other records that leave the site will be identified only by the Study Identification Number (SID) to maintain subject confidentiality. All records will be kept in a locked file cabinet. All computer entry and networking programs will be done using SIDs only. Clinical information will not be released without written permission of the subject, except as necessary for monitoring by IRB, the FDA, the NINDS, the OHRP, the sponsor, or the sponsor's designee. HIPAA guidelines for confidentiality and the principles of medical ethics will be adhered to during the study.

11.3 Study Modification/Discontinuation

The study may be modified or discontinued at any time by the IRB, the NINDS, the OHRP, or other government agencies as part of their duties to ensure that research subjects are protected.

The NIH, and local IRBs have the authority to stop or suspend this trial at any time. This study may be suspended or closed if:

- Accrual has been met
- The study objectives have been met

- The Study Chair / Study Investigators believe it is not safe for the study to continue • The NIH or FDA suspends or closes the trial

12 PUBLICATION OF RESEARCH FINDINGS

Publication of the results of this trial will be governed by the policies and procedures developed by the Executive Committee. Any presentation, abstract, or manuscript will be made available for review by the sponsor and the NINDS prior to submission.

13 REFERENCES

1. Towfighi A, Markovic D, Ovbiagele B. Half the Stroke Survivors in the United States have Poorly Controlled Hypertension. . *Stroke* 2012;43:A3356.
2. Cheng E, Chen A, Vassar S, Lee M, Cohen SN, Vickrey B. Comparison of secondary prevention care after myocardial infarction and stroke. *Cerebrovasc Dis.* 2006;21(4):235-241.
3. Cheng EM, Jolly D, Jones LA, Cohen SN. Modest improvement in risk factor control after admission for a stroke or transient ischemic attack. *J Stroke Cerebrovasc Dis.* Jul-Aug 2005;14(4):174-178.
4. Towfighi A, Saver JL. Stroke declines from third to fourth leading cause of death in the United States: historical perspective and challenges ahead. *Stroke.* Aug 2011;42(8):2351-2355.
5. Kelly-Hayes M, Beiser A, Kase CS, Scaramucci A, D'Agostino RB, Wolf PA. The influence of gender and age on disability following ischemic stroke: the Framingham study. *J Stroke Cerebrovasc Dis.* MayJun 2003;12(3):119-126.
6. Roger VL, Go AS, Lloyd-Jones DM, et al. Heart disease and stroke statistics--2012 update: a report from the American Heart Association. *Circulation.* Jan 3 2012;125(1):e2-e220.
7. Egan BM, Zhao Y, Axon RN. US trends in prevalence, awareness, treatment, and control of hypertension, 1988-2008. *Jama.* May 26 2010;303(20):2043-2050.
8. Furie KL, Kasner SE, Adams RJ, et al. Guidelines for the prevention of stroke in patients with stroke or transient ischemic attack: a guideline for healthcare professionals from the american heart association/american stroke association. *Stroke.* Jan 2011;42(1):227-276.
9. O'Donnell MJ, Xavier D, Liu L, et al. Risk factors for ischaemic and intracerebral haemorrhagic stroke in 22 countries (the INTERSTROKE study): a case-control study. *Lancet.* Jul 10 2010;376(9735):112-123.
10. Whitworth JA. 2003 World Health Organization (WHO)/International Society of Hypertension (ISH) statement on management of hypertension. *J Hypertens.* Nov 2003;21(11):1983-1992.
11. Lewington S, Clarke R, Qizilbash N, Peto R, Collins R. Age-specific relevance of usual blood pressure to vascular mortality: a meta-analysis of individual data for one million adults in 61 prospective studies. *Lancet.* Dec 14 2002;360(9349):1903-1913.
12. Mancia G, De Backer G, Dominiczak A, et al. 2007 Guidelines for the Management of Arterial Hypertension: The Task Force for the Management of Arterial Hypertension of the European Society of Hypertension (ESH) and of the European Society of Cardiology (ESC). *J Hypertens.* Jun 2007;25(6):1105-1187.
13. Rashid P, Leonardi-Bee J, Bath P. Blood pressure reduction and secondary prevention of stroke and other vascular events: a systematic review. *Stroke.* Nov 2003;34(11):2741-2748.
14. Lloyd-Jones DM, Hong Y, Labarthe D, et al. Defining and setting national goals for cardiovascular health promotion and disease reduction: the American Heart Association's strategic Impact Goal through 2020 and beyond. *Circulation.* Feb 2 2010;121(4):586-613.
15. Cheng EM, Asch SM, Brook RH, et al. Suboptimal control of atherosclerotic disease risk factors after cardiac and cerebrovascular procedures. *Stroke.* Mar 2007;38(3):929-934.
16. Morgenstern LB, Smith MA, Lisabeth LD, et al. Excess stroke in Mexican Americans compared with non-Hispanic Whites: the Brain Attack Surveillance in Corpus Christi Project. *Am J Epidemiol.* Aug 15 2004;160(4):376-383.
17. Rosamond W, Flegal K, Furie K, et al. Heart disease and stroke statistics--2008 update: a report from the American Heart Association Statistics Committee and Stroke Statistics Subcommittee. *Circulation.* Jan 29 2008;117(4):e25-146.
18. Qureshi AI, Mendelow AD, Hanley DF. Intracerebral haemorrhage. *Lancet.* May 9 2009;373(9675):1632-1644.

19. Table 30: Leading causes of death and numbers of death, by sex, race, and Hispanic origin: United States, 1980 and 2005. *http://www.cdc.gov/omhd/populations/AsianAm/AsianAm.htm#4* 2008. Accessed July 22, 2010.

20. 2002 heart and stroke statistical update. *Dallas (TX): American Heart Association.* 2001 2002.

14 APPENDICES APPENDIX I

... 28

APPENDIX I. Informed Consent

University of California, Los Angeles
Department of Health Services, Department of Neurology
INSTITUTIONAL REVIEW BOARD

PATIENT CONSENT
TO PARTICIPATE IN RESEARCH

Research Proposal: Secondary stroke prevention by Uniting Community and Chronic care model teams Early to End Disparities: the SUCCEED trial

Principal Investigator: Barbara Vickrey, MD, MPH
Professor and Vice Chair of the Department of Neurology in the David Geffen School of Medicine (DGSOM) at UCLA
710 Westwood Plaza C-109
Los Angeles, CA 90095
(310) 206-7671

Co-Investigator: Dr. Amytis Towfighi
Clinical Assistant Professor
Neurology Department
Rancho Los Amigos National Rehabilitation Center
(562) 401-7611

SITE PI **SITE**

You are asked to participate in a research study conducted by Drs. Vickrey, **[OTHER PI'S]** from University of California, Los Angeles (UCLA) and by site PI at site. This study is funded by the National Institute of Health. You were selected as a possible participant in this study because you have suffered a stroke in the last 90 days, you received care at this site, and need to improve control of at least one stroke risk factor. Your participation in this research study is entirely voluntary. Please read the information below, and ask questions about anything that you do not understand, before deciding whether or not to participate.

DISCLOSURE STATEMENT

Your health care provider may be an investigator of this research protocol, and as an investigator, is interested in both your clinical welfare and in the conduct of this study. Before entering this study or at any time during the research, you may ask for a second opinion about your care from another doctor who is in no way associated with this project. You are not under any obligation to participate in any research project offered by your physician.

PURPOSE OF THE STUDY

The purpose of this study is to test whether an outpatient care program called SUCCEED reduces the risk of a future stroke among persons with a recent stroke and improves their ability to care for their illness and measures the cost of conducting such a program.

PROCEDURES

If you volunteer to participate in this study, we would ask you to do the following:

- Complete this informed consent process today
- Participate in a brief 10 minute examination, including taking your blood pressure, height, weight and waist measurement, and 45 minute survey today
- Participate in a brief 5 minute examination, including taking your blood pressure, height, weight, and waist measurement, and 30 minute survey 3 months and 12 months from now
- Obtain a fingerstick blood check 3 months and 12 months from now. You will have your finger pricked to obtain approximately 10 drops of blood in order to examine your cholesterol level, blood sugar and other indicators of cardiovascular health. This will take approximately 5 minutes.
- Participate in a brief telephone survey 8 months from now
- Allow us to examine information about you in the hospital database about your stroke medications, cholesterol levels, and stroke related visits for the next 12 months
- If you are randomly assigned to the "SUCEED" intervention, you will be asked to participate in visits with a care manager and a community health worker to coordinate your stroke care and participate in educational sessions.
- If you are randomly assigned to the control arm, you will be mailed education materials that address the same topics discussed in the and telephone calls
- After the 12 months of the study is completed, you will be contacted every 6 months for up to 2 years to check for major health events such as stroke, TIAs, heart attack or death.

POTENTIAL RISKS AND DISCOMFORTS

The risk of participating in this research study is considered minimal. Your privacy will be protected as stated in the section below. It is possible that the finger prick will be associated with slight pain and that a bruise may develop at the site of the prick. In rare cases, fainting and infection may occur. This research study may involve risks that are currently unforeseeable. If you are randomly assigned to the "SUCCEED" intervention arm, you may feel uncomfortable during home visits. These visits are voluntary and you can decide not to participate in these visits.

POTENTIAL BENEFITS TO SUBJECTS

You may learn about stroke and ways to reduce the risk of having another stroke. You may also learn about how to self-manage your chronic illnesses. If you are randomly assigned to the intervention arm you may also benefit from community interaction with your peers. You may also benefit from having your stroke risk factors monitored during the study period.

ANTICIPATED BENEFITS TO SOCIETY

The results of this study will aid in designing cost-effective programs of stroke prevention for patients in Los Angeles County medical centers. The results of this study may also help design cost-effective programs to help educate patients on how to manage their illnesses.

PAYMENT FOR PARTICIPATION

To compensate you for your time, you will be paid for different encounters with the research team. You will receive up to $120 for your participation:

- $30 for participating in the examination and survey today
- $30 for participating in the survey and obtaining your blood pressure, body measurements and blood tests 3 months from now

- $30 for participating in the telephone survey 8 months from now
- $30 for participating in the survey and obtaining your blood pressure, body measurements and blood tests 12 months from now

FINANCIAL OBLIGATION
There is no financial obligation of the subject.

PRIVACY AND CONFIDENTIALITY
Only the members of the research team and those who participate in the same group with you will know that you are a research subject. No information about you, or provided by you during the research study will be disclosed to others without your written permission except:

1) If necessary to protect your rights or welfare (for example, if you are injured and need emergency care); or
2) If required by law (for example, elder abuse)

With the exception of the written consent form and the administrative data, all materials with collected information will use only a code number and will not include any personal identifying information. Your consent forms, answers to questions, and medical data will be kept in a locked file drawer within a locked room with access permitted only to the Principal and co-Investigators. Study code numbers will replace any personal identifying information.

Authorized representatives of the UCLA Office for Protection of Research Subjects or the National Institute of Health may need to review records of individual subjects. As a result, they may see your name; but they are bound by rules of confidentiality not to reveal your identity to others.

When the results of the research are published or discussed in conferences, no information will be included that would reveal your identity.

PARTICIPATION AND WITHDRAWAL
Your participation in this research is VOLUNTARY. If you choose not to participate, your choice will not affect your relationship with SITE, nor will it affect your right to health care or other services to which you are otherwise entitled. If you decide to participate, you are free to withdraw your consent and discontinue participation at any time without prejudice to your future care at SITE.

You will be paid for the portions of the study that you have completed.

WITHDRAWAL OF PARTICIPATION BY THE INVESTIGATOR
If you do not feel well enough to complete the study, it may be necessary for the Investigator to withdraw you from participating in this research. You will still be paid for the portions of the study that you completed.

NEW FINDINGS
During the course of the study, you will be informed of any significant new findings (either good or bad), such as changes in the risks or benefits resulting from participation in the research or new alternatives to participation that might cause you to change your mind about continuing in the study. If new information is provided to you, your consent to continue participating in this study will be re-obtained.

USE OF DATA FOR FUTURE RESEARCH

In the future, data collected for this study may be shared with other researchers for other studies that are unknown at this time. Any data shared with other researchers, will not include your name or other personal identifying information.

1. My data may be kept for use in future research to learn about, prevent or treat other health-related problems (for example: diabetes, Alzheimer's disease, or heart disease).

YES NO

CONTACT FOR FUTURE RESEARCH

UCLA researchers may contact me in the future to ask me to take part in other research studies.

YES NO

IDENTIFICATION OF INVESTIGATORS

If you have any questions or concerns about the research, please contact the Principal Investigator from UCLA, Dr. Barbara Vickrey at 310-206-7671. You may also contact the co-Investigators from [Insert PI contact information]. [Insert site PI contact information]

RIGHTS OF RESEARCH SUBJECTS

You may withdraw your consent at any time and discontinue participation without penalty. You are not waiving any legal rights because of your participation in this research study.

If you have questions regarding your rights as a research subject, contact the SITE IRB, ADDRESS or the Office for Protection of Research Subjects, UCLA, 11000 Kinross Avenue, Suite 102, Box 951694, Los Angeles, CA 90095-1694, (310) 825-8714.

SIGNATURE OF RESEARCH SUBJECT OR LEGAL REPRESENTATIVE

I have read (or someone has read to me) and understand the information provided above. I have been given an opportunity to ask questions and all of my questions have been answered to my satisfaction. I have been given a copy of this form, as well as a copy of the Subject's Bill of Rights.

BY SIGNING THIS FORM, I WILLINGLY AGREE TO PARTICIPATE IN THE RESEARCH IT DESCRIBES.

____

Name of Subject

____

Name of Legal Representative *(if not applicable, delete signature lines and references to legal representation)*

____ _______________
Signature of Subject or Legal Representative Date

SIGNATURE OF INVESTIGATOR

I have explained the research to the subject or his/her legal representative, and answered all of his/her questions. I believe that he/she understands the information described in this document and freely consents to participate.

____
Name of Investigator

__________________________________ _________________________
Signature of Investigator Date (must be same as subject's)

SIGNATURE OF WITNESS

My signature as witness certified that the subject or his/her legal representative signed this consent form in my presence as his/her voluntary act and deed.

____
Name of Witness

_________________________________ ________________________
Signature of Witness Date (must be same as subject's)
