# Supplementary material for: Effect of a Coordinated Community and Chronic Care Model Team Intervention vs Usual Care on Systolic Blood Pressure in Patients With Stroke or Transient Ischemic Attack: The SUCCEED Randomized Clinical Trial
Source: JAMA Netw Open. 2021 Feb 15;4(2):e2036227. doi: 10.1001/jamanetworkopen.2020.36227 (PMC7885035; doi:10.1001/jamanetworkopen.2020.36227)
Supplement: Supplement 3. — Data Sharing Statement [file jamanetwopen-e2036227-s003.pdf]

# Data Sharing Statement

Towfighi. Effect of a Coordinated Community and Chronic Care Model Team Intervention vs Usual Care on Systolic Blood Pressure in Patients With Stroke or Transient Ischemic Attack. *JAMA Netw Open*. Published February 15, 2021.
doi:10.1001/jamanetworkopen.2020.36227

**Data**
**Data available:** Yes
**Data types:** Deidentified participant data, Data dictionary
**How to access data:** https://www.ninds.nih.gov/Current-Research/Research-Funded-NINDS/Clinical-Research/Archived-Clinical-Research-Datasets
**When available:** Within 3 months of publication

**Supporting Documents**
**Document types:** None

**Additional Information**
**Who can access the data:** Researchers whose proposed use of data approved
**Types of analyses:** Specified purpose
**Mechanisms of data availability:** with a signed data access agreement
